# Endoplasmic reticulum stress regulates the intestinal stem cell state through CtBP2

**DOI:** 10.1038/s41598-021-89326-w

**Published:** 2021-05-10

**Authors:** Bartolomeus J. Meijer, Wouter L. Smit, Pim J. Koelink, Barbara F. Westendorp, Ruben J. de Boer, Jonathan H. M. van der Meer, Jacqueline L. M. Vermeulen, James C. Paton, Adrienne W. Paton, Jun Qin, Evelien Dekker, Vanesa Muncan, Gijs R. van den Brink, Jarom Heijmans

**Affiliations:** 1 Department of Gastroenterology and Hepatology, Amsterdam Gastroenterology and Metabolism, Tytgat Institute for Liver and Intestinal Research, Amsterdam UMC, University of Amsterdam, Meibergdreef 69-71, Amsterdam, The Netherlands; 2 Department of Molecular and Biomedical Science, Research Centre for Infectious Diseases, University of Adelaide, Adelaide, SA 5005 Australia; 3 Verna and Marrs McLean Department of Biochemistry and Molecular Biology, Department of Molecular and Cellular Biology, Alkek Center for Molecular Discovery, Baylor College of Medicine, Houston, TX 77030 USA; 4 Department of Gastroenterology and Hepatology, Amsterdam UMC, University of Amsterdam, Meibergdreef 9, Amsterdam, The Netherlands; 5 Roche Innovation Center Basel, F. Hoffmann-La Roche AG, Basel, Switzerland; 6 Department of Internal Medicine, Amsterdam UMC, University of Amsterdam, Meibergdreef 9, Amsterdam, The Netherlands

**Keywords:** Stem cells, Intestinal stem cells

## Abstract

Enforcing differentiation of cancer stem cells is considered as a potential strategy to sensitize colorectal cancer cells to irradiation and chemotherapy. Activation of the unfolded protein response, due to endoplasmic reticulum (ER) stress, causes rapid stem cell differentiation in normal intestinal and colon cancer cells. We previously found that stem cell differentiation was mediated by a Protein kinase R-like ER kinase (PERK) dependent arrest of mRNA translation, resulting in rapid protein depletion of WNT-dependent transcription factor c-MYC. We hypothesize that ER stress dependent stem cell differentiation may rely on the depletion of additional transcriptional regulators with a short protein half-life that are rapidly depleted due to a PERK-dependent translational pause. Using a novel screening method, we identify novel transcription factors that regulate the intestinal stem cell fate upon ER stress. ER stress was induced in LS174T cells with thapsigargin or subtilase cytotoxin (SubAB) and immediate alterations in nuclear transcription factor activity were assessed by the CatTFRE assay in which transcription factors present in nuclear lysate are bound to plasmid DNA, co-extracted and quantified using mass-spectrometry. The role of altered activity of transcription factor CtBP2 was further examined by modification of its expression levels using CAG-rtTA3-*CtBP2* overexpression in small intestinal organoids, sh*CtBP2* knockdown in LS174T cells, and familial adenomatous polyposis patient-derived organoids. *CtBP2* overexpression organoids were challenged by ER stress and ionizing irradiation. We identified a unique set of transcription factors with altered activation upon ER stress. Gene ontology analysis showed that transcription factors with diminished binding were involved in cellular differentiation processes. ER stress decreased CtBP2 protein expression in mouse small intestine. ER stress induced loss of CtBP2 expression which was rescued by inhibition of PERK signaling. CtBP2 was overexpressed in mouse and human colorectal adenomas. Inducible CtBP2 overexpression in organoids conferred higher clonogenic potential, resilience to irradiation-induced damage and a partial rescue of ER stress-induced loss of stemness. Using an unbiased proteomics approach, we identified a unique set of transcription factors for which DNA-binding activity is lost directly upon ER stress. We continued investigating the function of co-regulator CtBP2, and show that CtBP2 mediates ER stress-induced loss of stemness which supports the intestinal stem cell state in homeostatic stem cells and colorectal cancer cells.

## Introduction

Intestinal stem cells continuously self-renew and give rise to transit-amplifying (TA) cells that have lost self-renewal capacity but cycle for a number of times before exiting the cell cycle and undergoing terminal differentiation^1,2^. Intestinal stem cell fate is defined by WNT signaling, and mutations that result in unrestricted activation of WNT signaling cause stem cell accumulation and development of colorectal adenomas and cancer^3–5^. WNT signaling is essential for stem cell maintenance, but mechanisms that regulate loss of self-renewal capacity and induce the transition from the stem cell to the TA cell state, remain poorly defined.

Intestinal stem cells are remarkably sensitive to endoplasmic reticulum (ER) stress, and exposure to ER stress triggers the rapid transition to the TA cell state in vitro and in vivo^6,7^. ER stress is a form of proteotoxic stress perceived by a cell when the ER folding capacity is overloaded and misfolded proteins accumulate, resulting in recruitment of the ER-resident transmembrane chaperone Glucose-regulated protein 78 (GRP78). This releases the sensor domains of specialized ER transmembrane receptors from the inhibitory interaction of GRP78, triggering a multi-layered signaling cascade known as the unfolded protein response (UPR). Intestinal stem cell differentiation is dependent on activation of protein kinase R (PKR)-like endoplasmic reticulum kinase (PERK) and consequent phosphorylation of Eukaryotic translation initiation factor 2A (eIF2α)^8^. This PERK-eIF2α pathway is an adaptive UPR effector pathway that regulates transient and immediate attenuation of global mRNA translation in order to alleviate ER protein load and resolve acute stress. This implicates that inhibition of global translation is a potential trigger for stem cell differentiation. Indeed, stem cell differentiation could be induced by phosphorylation of eIF2α using salubrinal^7^. Similar sensitivity to ER stress was observed in hematopoietic stem cells and in the squamous epithelium of the esophagus^9,10^.

In addition to stem cell homeostasis, intervening with the UPR induces differentiation of *Apc* mutant adenomatous stem cells and cancer cells that maintain self-renewal capacity within tumors, which leads to suppression of tumor formation^7,11,12^. Upon deletion of intestinal *Grp78*, differentiation of *Apc* mutant stem cells occur whilst nuclear translocation of ß-catenin remains intact^11^. This implies that the trigger for differentiation may occur downstream—and in the presence of—oncogenic activation of the WNT signaling effector ß-catenin.

The relationship between translational regulation of proteins and stem cell differentiation remains unclear. However, several transcription factors that are required for proper stem cell fate have a remarkably short protein half-life, consistent with predominance of translational control over gene expression^13,14^. Thus, presence and function of these proteins dependents on continuous translation and may be significantly affected by transient translation attenuation due to ER stress^15^. In this way, depletion of such factors by a translational arrest could contribute to induce the transition from stemness to a differentiated state. One short-lived transcription factor important for stem cell homeostasis is c-MYC, downstream of ß-catenin^16^. ER stress results in a rapid and PERK-eIF2α dependent depletion of c-MYC^6,7^. We hypothesized that PERK-eIF2α-induced inhibition of global translation results in depletion of short-lived transcription factors, such as c-MYC, that may play a key role in maintenance of the intestinal stem cell state.

Aiming to discover novel regulators of the intestinal stem cell state, which could expose potential targets to sensitize cancer stem cells for chemo- and radiotherapy, we here used a mass spectrometry-based screening method to quantify DNA-binding activity of transcription factors to their binding sites in order to identify novel transcriptional regulators that rapidly lose their activity in response to ER stress.

## Results

### An unbiased proteomics screen reveals a set of transcription factors that show altered DNA binding in response to ER stress

**Figure 1:**
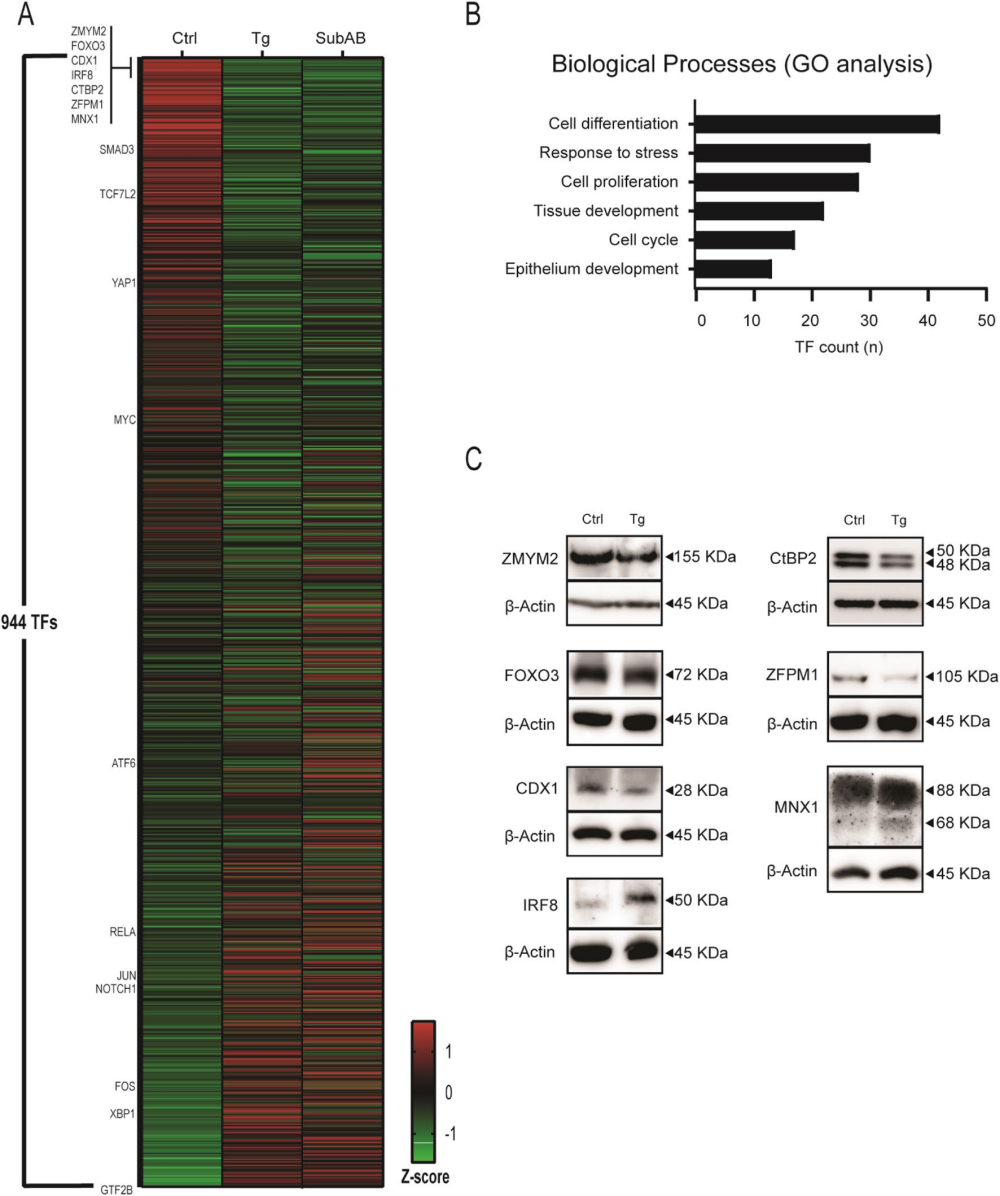
ER stress induced loss of stemness is characterized by altered activity of a set of transcription factors. (**A**) Heatmap based on average Z-scores of performed CatTFRE assay on human LS147T cells treated for 2 h with DMSO (n = 3) thapsigargin (n = 3) or SubAB (n = 2). Upper selection contains transcription factors with most downregulated binding to DNA template. (**B**) DAVID gene ontology (GO) analysis on top 100 most downregulated transcription factors, presented in the heatmap. (**C**) Protein analysis by western blot of the heatmap selected transcription factors, in LS174T cells, 24 h after thapsigargin treatment. Full-length blots/gels are presented in Supplementary Immunoblotting Data Figure 1. All western blot images were generated and exported using ImageQuant LAS 4000 software and cropped and labeled using Adobe Illustrator software version 25.2.

When intestinal stem cells are exposed to chemically induced ER stress, consequent activation of the UPR determines their cellular fate by driving a process of differentiation while abrogating programs that drive stem cell fate. This occurs in a PERK-eIF2α-dependent manner^6,11,12^. To identify which transcription factors that drive stemness are lost in the early phase of the ER stress response, even before cells have committed to a differentiation program, we subjected LS174T colorectal cancer cells for 2 h to 400 nM of thapsigargin. We used LS174T cells since these cells, which express a mutant form of β-catenin, possess a thoroughly characterized intestinal epithelial stem cell signature^4^. Furthermore, we have previously used this cell line to describe genotypic and phenotypic dynamics of loss of stemness following chemically induced ER stress^6,11,12^. Since analysis of transcription factors using proteomics is hampered by the low abundance of these proteins, we enriched for transcription factors with DNA-binding activity prior to protein analysis using the catTFRE assay^17^. After 2 h of thapsigargin treatment, we observed the expected ER stress response in these cells, with phosphorylation of eIF2α (Figure S1A) and increased transcriptional expression of the immediate-early response genes *GRP78*, *XBP1(s)* and *CHOP* (Figure S1B). We included a second ER stress condition in which we treated cells for 2 h with subtilase cytotoxin (SubAB), a bacteria-derived toxin that specifically cleaves the essential ER-resident chaperone GRP78, in order to capture the early phase of UPR activation which is caused by disturbed protein folding that triggers a robust ER stress response (Figure S1D)^18–20^. Following these immediate-early response genes, several downstream UPR targets associated with the X-box binding protein 1 (XBP1) and Activating transcription factor 6 (ATF6) branch were upregulated in both treatment groups, indicating a fully elicited UPR at these later timepoints (Figure S1C and E). After 2 h of exposure to ER stress, we extracted the nuclear protein lysate and examined for binding of transcription factors to a DNA template, consisting of ~ 1000 transcription factor responsive elements (Figure S1F). Over 900 transcription factors bound to plasmid DNA could be measured by mass spectrometry (Fig. 1A). Amongst the transcription factors with increased binding activity were well-known ER stress response targets including XBP1, c-JUN and c-FOS^21^. Gene ontology analysis of the top 100 transcription factors with reduced DNA-binding activity showed that a significant number of transcription factors were involved in biological processes such as cell differentiation and response to stress (Fig. 1B). Based on our hypothesis that transcription factors essential for stemness are lost upon the PERK-eIF2α-dependent global translation arrest, we focused on a subset of the transcription factors with reduced DNA-binding activity that were present in both treatment groups. First, we confirmed reduced protein expression of several transcription factors after 24 h of ER stress using immunoblot to show that the proteins with diminished DNA-binding activity were also depleted in term of cellular abundance as a result of translational arrest. Interestingly, lower DNA-binding activity in the catTFRE assay did not necessarily correspond with a reduction of cellular protein abundance 24 h after induction of ER stress (Fig. 1A, C). One of the transcription factors that was most significantly downregulated in both treatment groups was C-terminal-binding protein 2 (CtBP2). CtBP2 does not directly bind to DNA, but functions as a pleiotropic co-regulator that is able to bind and regulate the activity of several transcription factor complexes^22,23^. Furthermore, CtBP2 has been described to be overexpressed in several human cancers including CRC, and is involved in oncogenic hallmarks including cancer cell survival and migration^24^. Using an unbiased approach identifying low abundance nuclear transcription factors that are lost shortly upon induction of ER stress, we identify CtBP2 and further characterized the role of this transcriptional regulator on the intestinal stem cell program.

### CtBP2 is predominantly localized in stem cells of the small intestinal epithelial crypt where its protein expression is affected by ER stress

**Figure 2:**
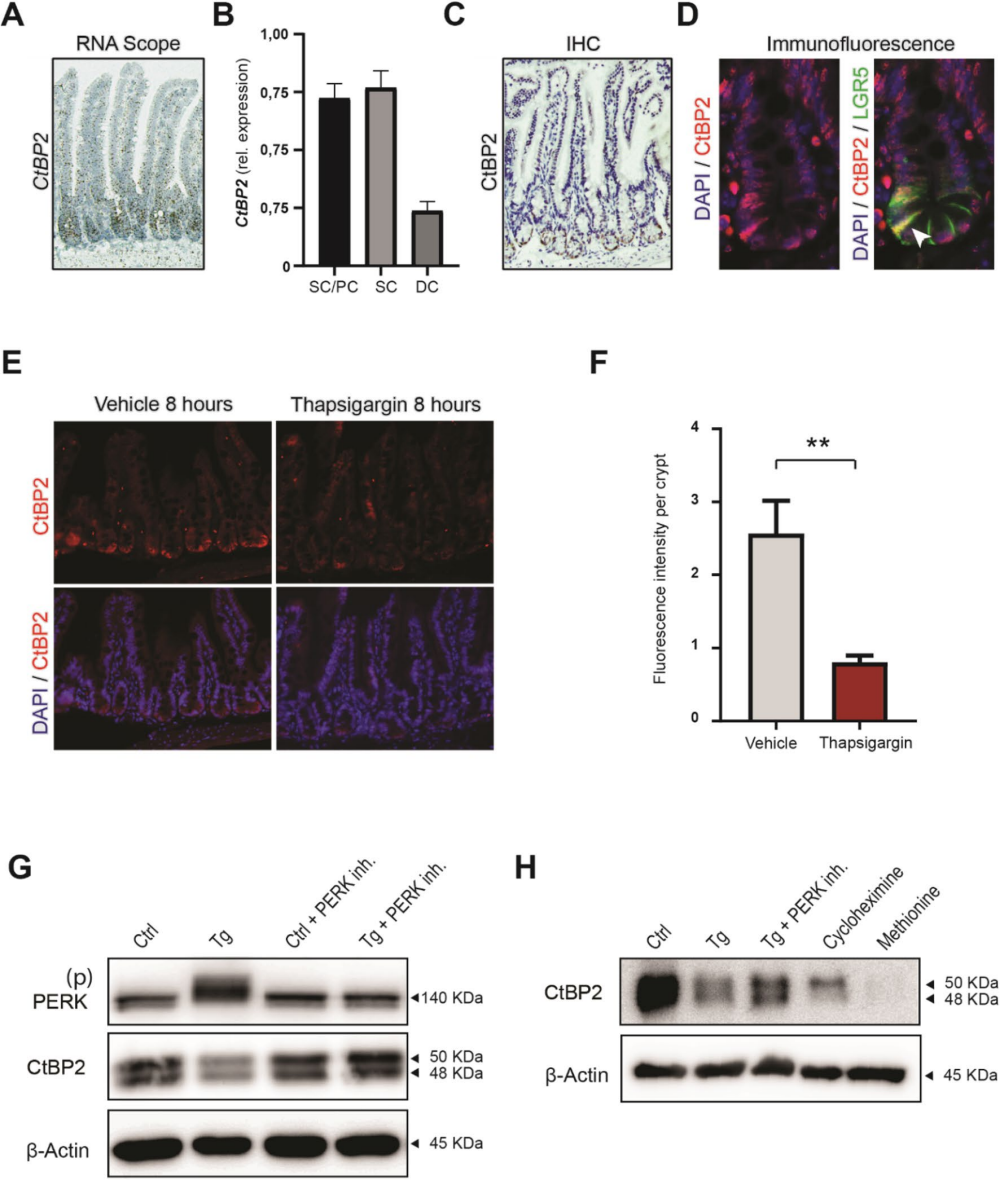
CtBP2 is predominantly localized in the small intestinal epithelial crypt and is involved in ER stress induced differentiation. (**A**) Representative images of mouse small intestinal RNA scope in situ hybridization for *Ctbp2* mRNA during homeostasis. (**B**) RT-qPCR for *Ctbp2* in flow cytometry sorted epithelial cells, derived from mouse small intestine. Sorted for stem/Paneth cells (SC/PC); Epcam^high^CD45^low^CD24^high^, stem cells (SC); Epcam^high^CD45^low^CD24^med^ and differentiated cells (DC); Epcam^high^CD45^low^CD24^low^. (**C**) Immunohistochemistry for CtBP2 in small intestine (n = 4). (**D**) Immunofluorescence for CtBP2 (red) in small intestine of a *Lgr5*-EGFP-IRES-creERT2 knock-in (green) mouse with DAPI (blue) counterstain (n = 4). White arrow indicates CTBP2 positive stem cell. (**E**) Representative image of immunofluorescence for CtBP2 (red) in small intestine of mice treated for 8 h with DMSO vehicle or thapsigargin, with DAPI (blue) counterstain. (**F**) Quantification of the fluorescence intensity in the small intestine of DMSO or thapsigargin treated mice (n = 6), at least 20 crypts evaluated per mouse. (**G**) Representative western blot for PERK and CtBP2 in human LS174T cells treated (n = 2) with 400 nM thapsigargin for 24 h in the presence or absence of 400 nM Perk inhibitor. Full-length blots/gels are presented in Supplementary Immunoblotting Data Figure 2. (**H**) Representative experiment showing expression of newly synthesized CtBP2 protein by *L*-azidohomoalanine labeling and pulldown of labeled nascent proteins following 4 h of 400 nM thapsigargin in the presence or absence of 400 nM Perk inhibitor. Cycloheximide (100 mM) or 50:50 ratio of methionine and *L*-azidohomoalanine were used as negative controls for protein synthesis and specific labeling respectively (n = 3). Full-length blots/gels are presented in Supplementary Immunoblotting Data Figure 3. All immunohistochemistry images were captured with a Leica DM6000 microscope using LAS AF software (Leica, Wetzlar, Germany). All western blot images were generated and exported using ImageQuant LAS 4000 software and cropped and labeled using Adobe Illustrator software version 25.2. Graph bars show mean and s.e.m. **P < 0.01 (Student’s *t*-test). *Ctrl* control, *Tg* thapsigargin.

To further understand the role of CtBP2 in the intestinal epithelium, we first identified which cells express CtBP2 in healthy small intestinal epithelium. We assessed *Ctbp2* mRNA expression in the mouse intestine by in situ hybridization (RNAscope), which revealed high expression in the crypt base and TA-zone where stem cells and proliferating cells reside respectively, whereas only marginal expression was found in the villus (Fig. 2A). Quantitative RT-PCR analysis on flow cytometry sorted stem cells (Epcam^high^CD45^low^CD24^med^), Paneth cells (Epcam^high^CD45^low^CD24^high^) and differentiated enterocytes (Epcam^high^CD45^low^CD24^low^) of the small intestinal epithelium confirmed that *Ctbp2* expression was high in stem- and Paneth cells when compared to differentiated epithelial cells (Fig. 2B, S2A and B). However, it must be noted that the isolated Paneth cell population also contained *Lgr5* positive cells, as complete separation between these compartments could not be fully achieved. We next examined CtBP2 protein expression by immunohistochemistry, which showed CtBP2 expression mainly in the mouse intestinal crypt region (Fig. 2C). Co-localization of CtBP2 and eGFP in immunofluorescent staining of *Lgr5-*EGFP-IRES-creERT2 mice, in which stem cells are marked by eGFP expression, further supported that stem cells express CtBP2, suggesting a role in the regulation of stemness. Expression was also observed in other cells of the crypt, predominantly in Paneth cells (Fig. 2D).

To demonstrate that CtBP2 protein expression in the intestinal crypt bottom is affected by ER stress in vivo, we administered intraperitoneal injection of thapsigargin or vehicle (DMSO) in adult mice and assessed small intestinal tissue 8 h after administration. Although *Ctbp2* mRNA levels were not significantly altered (Figure S3A), CtBP2 protein expression in the crypt was markedly lower (Fig. 2E, F). This implicates that intestinal CtBP2 is mainly regulated post-transcriptionally during exposure to ER stress. In line with diminished CtBP2 downstream activity, mRNA expression of T-cell lymphoma invasion and metastasis 1 (*Tiam1*), one of the few described targets directly activated by CtBP2 at the transcriptional level, was significantly downregulated in these mice (Figure S3B). Induction of ER stress with the highly specific ER-stress inducing agent SubAB in the human CRC cell line LS174T also lowered *TIAM1* expression (Figure S3C). Next, we analyzed if loss of CtBP2 protein upon induction of ER stress was dependent on PERK-eIF2α signaling. To this end we treated LS174T cells with thapsigargin and specifically inhibited PERK activation using a specific PERK inhibitor (GSK2606414). Indeed, we observed that induction of ER stress for 24 h led to loss of CtBP2 expression that could be largely rescued upon inhibition of eIF2α signaling, indicating that CtBP2 protein levels are translationally regulated by PERK-eIF2α signaling (Fig. 2G). A similar result was found when ER stress was induced using tunicamycin, a naturally occurring antibiotic that induces ER stress in cells by interfering with protein folding (Figure S3D). To demonstrate that ER stress results in rapid PERK-dependent loss of nascent CtBP2 protein, we assessed CtBP2 expression following pulldown of nascent protein using *L*-azidohomoalanine (AHA) labeling. As expected, 4 h of thapsigargin resulted in near complete loss CtBP2 synthesis that could be largely rescued when PERK was inhibited (Fig. 2H). Thus, our data indicates that intestinal CtBP2 expression strongly depends on continuous global protein translation, causing a proclivity for rapid loss of expression following the translational pause as part of the activated UPR during ER stress.

### CtBP2 regulates stemness in mouse small intestinal epithelial organoids

**Figure 3:**
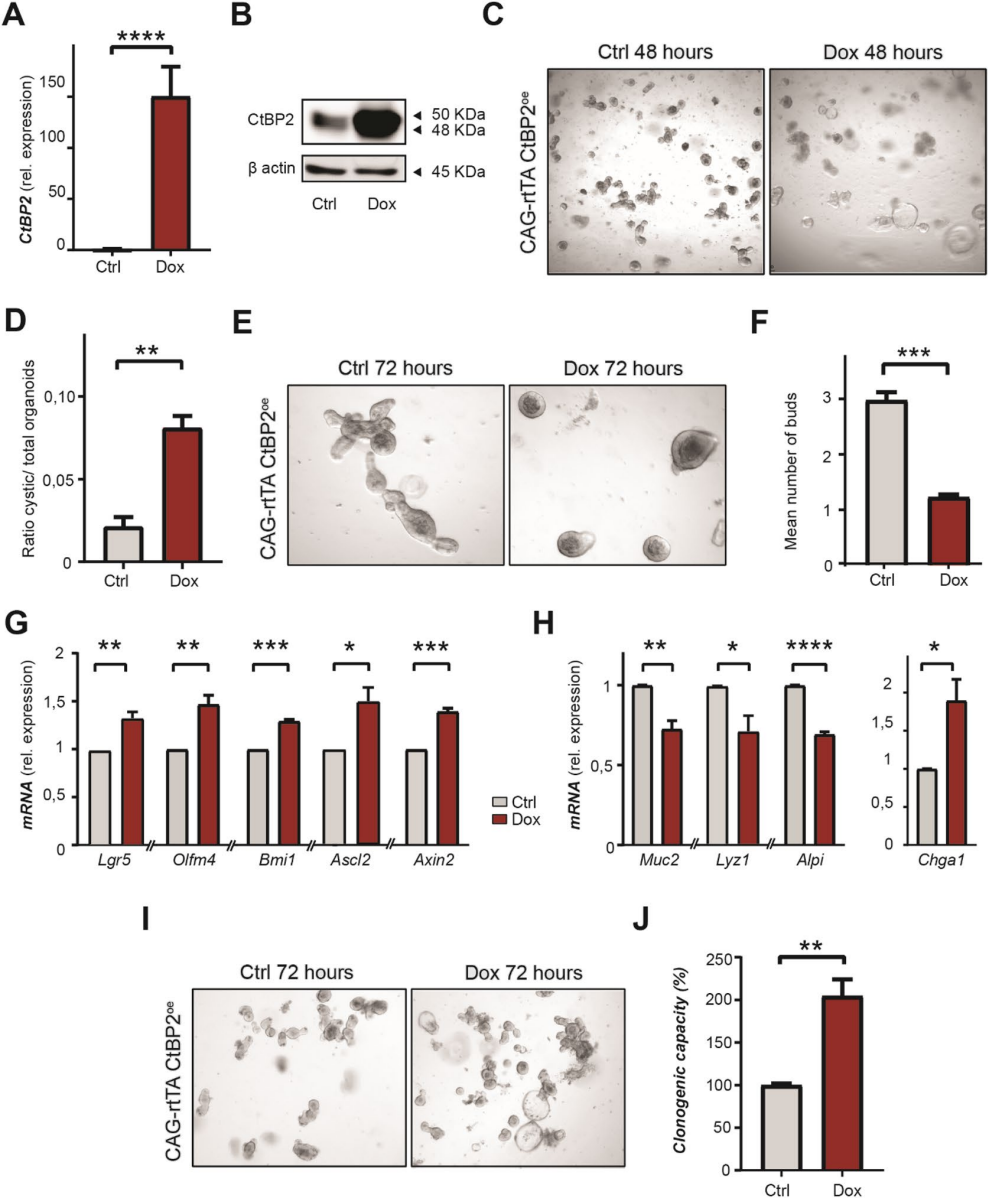
CtBP2 regulates stemness in mouse small intestinal epithelial organoids. (**A**) RT-qPCR of *CtBP2* in wildtype or *CtBP2* overexpressed mouse small intestinal organoids 48 h after doxycycline-induced *CtBP2* overexpression (n = 3). (**B**) CtBP2 protein expression by western blot in CAG-rtTA-*CtBP2* organoids, 48 h after treatment with doxycycline. Full-length blots/gels are presented in Supplementary Immunoblotting Data Figure 4. (**C**) Representative image of CAG-rtTA-*CtBP2* mouse small intestinal organoids 48 h after doxycycline-induced overexpression. (**D**) Quantified ratio of cystic organoids to normal organoids 48 h after doxycycline-induced CtBP2 overexpression in (n = 3). (**E**) Representative images of budding in CAG-rtTA-*CtBP2* mouse small intestinal organoids, 72 h upon doxycycline-induced CtBP2 overexpression. (**F**) Mean number of buds per organoid in *CtBP2* wildtype (non-induced) versus doxycycline-induced CtBP2 overexpression organoids (n = 3). (**G**) RT-qPCR of stem cell markers and (**H**) differentiation markers in CAG-rtTA-*CtBP2* mouse small intestinal organoids 72 h after doxycycline-induced CtBP2 overexpression (n = 3). (**I**) Representative images of outgrowth of induced CAG-rtTA-*CtBP2* organoids 72 h after single cell seeding. Images were captured with a Leica DM6000 microscope using LAS AF software (Leica, Wetzlar, Germany). All western blot images were generated and exported using ImageQuant LAS 4000 software and cropped and labeled using Adobe Illustrator software version 25.2. (**J**) Calculated clonogenic capacity (%) of induced CAG-rtTA-*CtBP2* organoids after single cell seeding. Graph bars show mean and s.e.m. *P < 0.05, **P < 0.01, ***P < 0.001, ****P < 0.0001 (Student’s *t*-test).

Next, we investigated the effect of CtBP2 activity on the regulation of intestinal stem cells and the ability of these cells to self-replicate and give rise to differentiated lineages. To this end we expressed the evolutionary conserved coding sequence of human *CtBP2* under the control of a tetracycline responsive promoter into mouse small intestinal organoids expressing the tetracycline responsive transcription factor rtTA3. By pretreating these organoids with a GSK3β inhibitor, we enriched for stem cells and ensured that the CtBP2 plasmid could be integrated in the stem cell genome^25^. After puromycin selection, the resulting non-induced organoids looked phenotypically as wildtypes. Administration of doxycycline resulted in robust overexpression of CtBP2 at mRNA and protein level (Fig. 3A, B). Strikingly, 48 h after passaging and induction of CtBP2 overexpression, organoids started to adopt a cystic morphology, a phenotype associated with predomination of proliferative cells (Fig. 3C, D)^26^. Over time, whereas wildtype control organoids started budding as a sign of normal differentiation into cells found on the villus of the small intestine, CtBP2 overexpression resulted in fewer and shorter buds per organoid indicative of perturbed differentiation (Fig. 3E, F). Quantitative RT-PCR analyses indeed revealed enrichment of stem cell markers at 72 h upon CtBP2 overexpression, as shown by upregulation of the stem cell markers *Lgr5*, *Olfm4* and *Bmi1* (Fig. 3G). Expression of *Ascl2* and *Axin2*, known regulators of WNT-associated genes fundamental to the stem cell state, were also significantly increased (Fig. 3G)^27^. Decreased transcriptional expression of lineage-specific differentiation makers indicated that the increased stem cell pool was at the cost of perturbed differentiation into Paneth cells, goblet cells, and enterocytes respectively (Fig. 3H). Of note, there was significant increase of chromogranin A (*Chga*) mRNA expression, suggesting persistence of the entero-endocrine lineage in these organoids (Fig. 3H). To functionally demonstrate that the self-renewal capacity was enhanced, we performed clonogenicity assays, testing the capacity of single stem cells to grow into fully developed organoids. CtBP2 overexpression indeed markedly increased the clonogenic capacity of single cells (Fig. 3I, J)^28^. Altogether, these results indicate that CtBP2 promotes the self-renewal capacity of intestinal stem cells.

### High levels of CtBP2 protect epithelial stem cells against ER stress and stress from ionizing radiation

**Figure 4:**
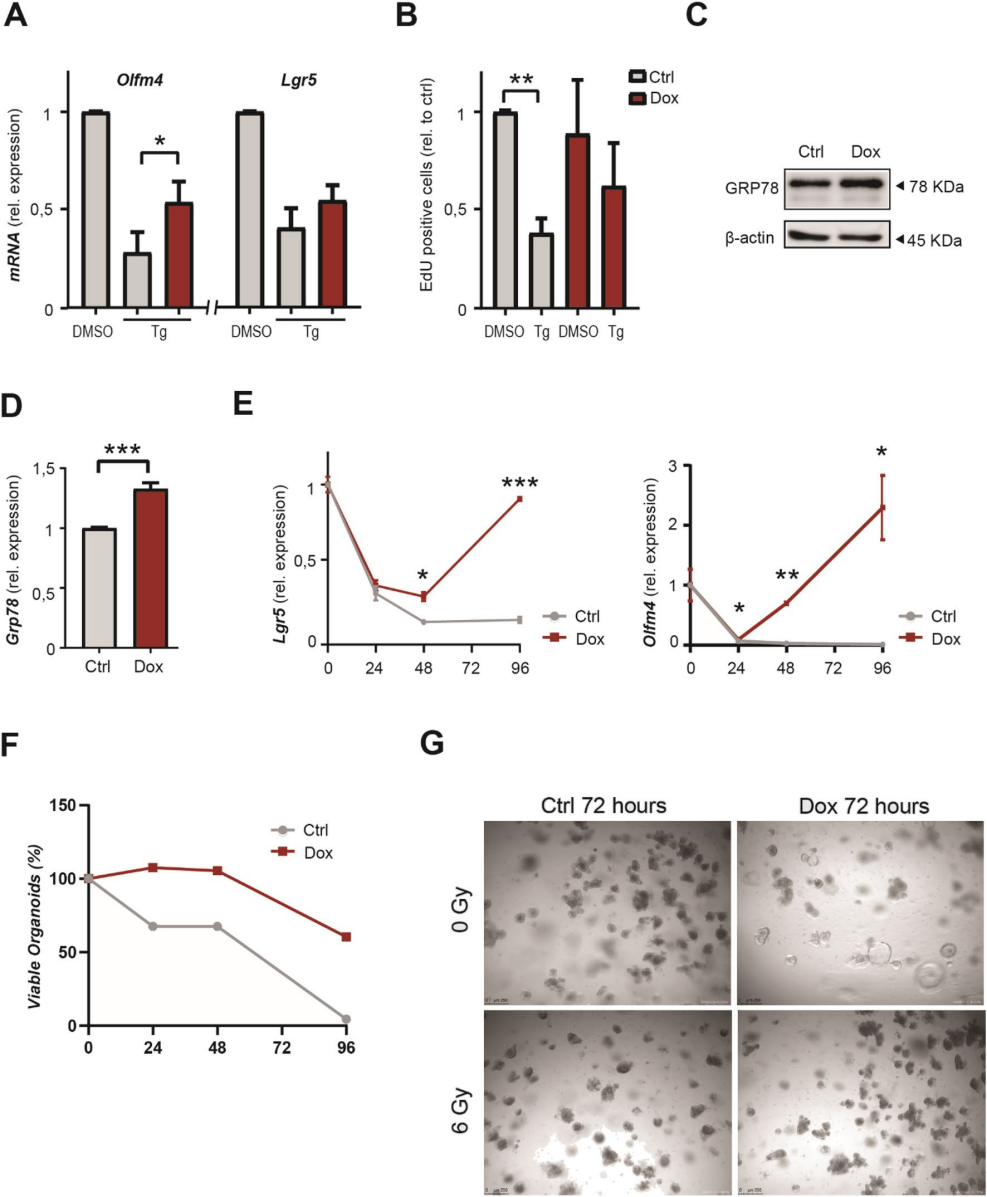
High levels of CtBP2 protect epithelial stem cells against ER stress and gamma irradiation. (**A**) RT-qPCR of stem cell markers in induced CAG-rtTA-*CtBP2* organoids after 6 h of DMSO or thapsigargin treatment (n = 2). (**B**) EdU incorporation in induced CAG-rtTA-*CtBP2* organoids 6 h after thapsigargin treatment (n = 3). (**C**) Western blot of GRP78 expression upon induction of CtBP2 overexpression. Full-length blots/gels are presented in Supplementary Immunoblotting Data Figure 5. (**D**) RT-qPCR of *Grp78* upon induction of CtBP2 overexpression (n = 3). (**E**) RT-qPCR of stem cell markers induced CAG-rtTA-*CtBP2* organoids during a time course following 6 Gy of irradiation (n = 3). (**F**) Quantification of viable organoids after 6 Gy irradiation (n = 2). (**G**) Representative images of the phenotype of induced CAG-rtTA-*CtBP2* organoids that were irradiated 72 h ago with 6 Gy (n = 3). Images of organoids were captured with a Leica DM6000 microscope using LAS AF software (Leica, Wetzlar, Germany). All western blot images were generated and exported using ImageQuant LAS 4000 software and cropped and labeled using Adobe Illustrator software version 25.2. Graph bars show mean and s.e.m. *P < 0.05, **P < 0.01, ***P < 0.001, (Student’s *t*-test or one-way ANOVA).

Preservation of stem cell integrity is a prerequisite for long-term maintenance of a functional stem cell pool and thereby restoration of tissue homeostasis following exposure to conditions of stress^29^. As a consequence, stress response pathways regulate the cellular stem cell fate, driving differentiation or apoptosis in order to prevent propagation of damaged stem cells^6,30^. However, stem cell factors that protect or increase resilience against the various forms of cellular stress are poorly defined. To assess the potential role of CtBP2 herein, we subjected intestinal organoids to proteotoxic and genotoxic stress, using chemical induction of ER stress or ionizing radiation respectively. As expected, 6 h of thapsigargin induced ER stress resulted in a strong reduction of *Lgr5* and *Olfm4* levels (Fig. 4A). This was also functionally reflected in diminished proliferation as measured by incorporation of 5-ethynyl-2′-deoxyuridine (EdU) (Fig. 4B). CtBP2 overexpression partially rescued these effects on stem cell marker expression and proliferation rates (Fig. 4A, B and S3F). Interestingly, we observed that baseline mRNA and protein levels of the major ER chaperone GRP78 was elevated when CtBP2 was overexpressed, which could lead to mitigation of the degree of ER stress in these cells by increasing the threshold for UPR activation (Fig. 4C, D)^31,32^. Induced CtBP2 expression did not elicit the UPR in itself, since we did not detect elevation of *Xbp1* splicing (Figure S3E). Lost CtBP2 protein levels during thapsigargin treatment were largely restored following CtBP2 overexpression, suggesting that CtBP2 mediated maintained stemness during ER stress (Figure S3F).

Next, we assessed a functional role for CtBP2 in self renewal and stem cell function by submitting organoids with CtBP2 overexpressing to radiation-induced injury. Gamma irradiation induces damage, leading to cell death and loss of proliferation of cells. Eventually a tissue repair response in which intestinal stem cells are known to be indispensable, results in tissue restauration^33^. To assess the stem cell dynamics of the response following irradiation injury, we measured *Lgr5* and *Olfm4* expression levels over the course of days. CtBP2 overexpression mitigated reduced mRNA expression of *Lgr5* and *Olfm4* and promoted restoration to normal levels (Fig. 4E). Strikingly, whilst wildtype organoids did not regenerate properly and mostly disintegrated days after irradiation, CtBP2 overexpression protected against this injury phenotype (Fig. 4F, G). Taken together, these findings support a role for CtBP2 in protection of the stem cell pool during conditions of proteotoxicity from ER stress and genotoxicity from ionizing radiation.

### CtBP2 is essential for clonal expansion of APC deficient cells

**Figure 5:**
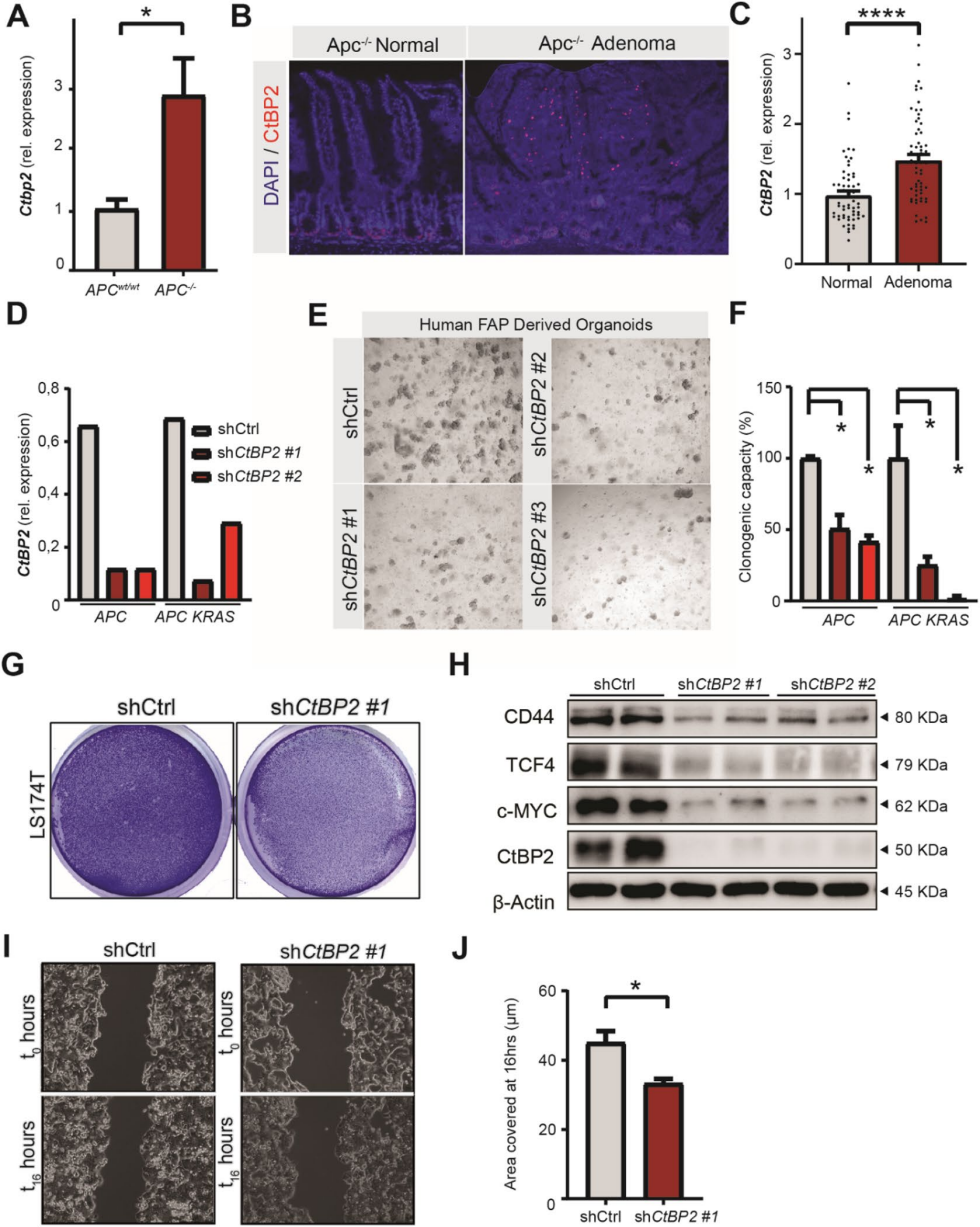
CtBP2 is important for clonal expansion of a WNT-high cancer cell line and colon organoids derived from FAP patients. (**A**) RT-qPCR of *Ctbp2* in small intestinal tissue of *Apc* homozygous mice (n = 4). (**B**) Immunofluorescence for CtBP2 (red) in *Apc* heterozygous adenoma, with DAPI (blue) counterstain. (**C**) RT-qPCR of *CtBP2* in adenomatous tissue of a patient cohort (n = 60 per group). (**D**) Knockdown efficiency measured by RT-qPCR of *CtBP2* in FAP patient-derived organoids carrying mutations in *APC* with and without additional mutations in *KRAS*. (**E**) Representative images showing outgrowth of FAP patient-derived organoids transduced with *shCtBP2*, 72 h after single cell seeding. (**F**) Clonogenic capacity of two FAP patient-derived organoids after knockdown of *CtBP2* (n = 2). (**G**) Representative image of crystal violet staining on LS174T cells 5 days after lentiviral transduction with *shCtrl* or *shCtBP2*. (**H**) WNT-associated proteins analyzed by western blot in LS174T cells transduced with *shCtrl* or *shCtBP2*. Full-length blots/gels are presented in Supplementary Immunoblotting Data Figure 6. (**I**) Representative image of migration scratch assay on LS174T cells 5 days after lentiviral transduction with *shCtrl* or *shCtBP2,* analyzed immediately after and 16 h after the scratch injury. (**J**) Quantification of covered area of migrating cells 16 h after the scratch injury (n = 3). Images of organoids were captured with a Leica DM6000 microscope using LAS AF software (Leica, Wetzlar, Germany). All western blot images were generated and exported using ImageQuant LAS 4000 software and cropped and labeled using Adobe Illustrator software version 25.2. Graph bars show mean and s.e.m. *P < 0.05, **P < 0.01, ***P < 0.001, ****P < 0.0001 (Student’s *t*-test or one-way ANOVA).

One of the hallmarks of cancer, including CRC, is sustained proliferation of cancer cells, which possess stem-like characteristics that are essential for tumor growth and maintenance^34^. In CRC, approximately 80% of sporadic cancers carry a loss-of-function mutation in the tumor suppressor gene *APC*, resulting in autonomous high WNT activity due to β-catenin stabilization, nuclear activation and TCF4-dependent gene expression^35^. This transcriptional signature instructs a hyperactivated stem cell state in colonic cells that drives the formation of adenocarcinomas^35^. To investigate the role of CtBP2 on stemness in APC deficient intestinal cells, we assessed whether high WNT activity relies on functional CtBP2. Using *Villin*-Cre-ERT2-*Apc*^580S/580S^ small intestinal organoids, we could delete *Apc* in a homozygous manner by 4-OH tamoxifen addition to the culture medium, leading to transformation of intestinal cells that resembles the early adenoma program. Upon induction by 4-OH tamoxifen, we found significant upregulation of *Ctbp2* (Fig. 5A). Furthermore, in *Apc* deficient adenomas that had developed in heterozygous *Apc* mutant mice, CtBP2 expression was high at the basolateral site of the adenomas, with hotspots in central and luminal areas (Fig. 5B), corresponding with the described distribution of Lgr5-positive tumor stem cells in early-stage adenomas^36^. To examine the relevance of CtBP2 in human adenomatous cells, we analyzed *CtBP2* expression of adenomatous tissue and matched normal adjacent colonic tissue in a cohort of 60 patients. As expected, *CtBP2* levels were significantly elevated in adenomas compared to healthy tissue, supporting a role for CtBP2 in human adenomagenesis (Fig. 5C). To show that depletion of CtBP2 functionally affects stemness in human adenoma cells with a mutation in *APC*, we made use of colonic epithelial organoids derived from adenomas of patients with familial adenomatous polyposis (FAP). These patients carry germline deletions in *APC* and therefore develop multiple intestinal adenomas early in life that arise from loss of heterozygosity. We cultured two *APC* deleted tumor-derived organoid lines, with and without a spontaneous mutation in the *KRAS* oncogene (abbreviated organoids line A and AK). We transduced these organoids with lentiviral constructs containing *shRNAs* against *CtBP2*, or a scrambled control short hairpin (*shCtrl*) (Fig. 5D)^25^. Notably, whereas *shCtrl* organoids could be easily propagated after transduction, *CtBP2* depletion strongly disturbed organoid outgrowth and viability (Fig. 5E). As expected, the self-renewal capacity as measured by the clonogenic single cells capacity to form organoids was significantly compromised in APC deficient cells, regardless of the presence of the *KRAS* oncogene (Fig. 5F). This suggests that CtBP2 is also crucial for stemness in APC deleted cell of adenomas from FAP patients, at least in vitro.

Finally, we investigated the effect of CtBP2 depletion in the LS174T CRC cell line, which is derived from a spontaneous Dukes B colon adenocarcinoma and has a well-described deregulated WNT/β-catenin oncogenic signature^37^. Transduction of *shRNAs* against *CTBP2* in these cells also resulted in strong downregulation of *CtBP2* (Figure S4A). *CTBP2* knockdown significantly inhibited cell growth and proliferation, assessed by crystal violet staining, EdU incorporation and reduction of stem cell markers (Fig. 5G, Figure S4B and C). Furthermore, protein expression of the WNT-associated transcription factors c-MYC, TCF4, and downstream target CD44 were completely lost after *CtBP2* knockdown, indicating that CtBP2 operates upstream or at the level of TCF4, which is consistent with a previous report (Fig. 5H)^38^. In addition, multiple intestinal stem cell markers were downregulated including *LGR5, CD44, LRIG1* and *BMI1* (Figure S4C) whilst differentiation markers *P21* and *MUC2* were increased (Figure S4D). Next to growth, cell migration which is another hallmark important for colon cancer progression associated with WNT signaling^39^, was also impaired by *CtBP2* knockdown (Fig. 5I, J). This fits with reported function of CtBP2 to promote cell migration through the downstream guanine nucleotide exchange factor (GEF) Tiam1^40^. Indeed, we found that *TIAM1* expression was lower in these cells (Figure S4E). Moreover, high *TIAM1* expression correlated with decreased overall survival in a large dataset of CRC patients (Figure S4F). Expression was particularly high in the CMS1 and CMS4 subtypes, which are the cancers with a poor prognostic outcome (Figure S4G).

Taken together, we demonstrate that ER stress induced loss of stemness is characterized by diminished activity of various transcription factors. One of these factors, co-regulator CtBP2, was found to be important for maintenance of intestinal stem cell function. Furthermore, our data supports a crucial role for CtBP2 in stemness and growth of adenomatous and carcinomatous cells that are characterized by an oncogenic WNT signature.

## Discussion

Cellular abundance of proteins is predominantly controlled at the level of mRNA translation^41^. Stem cells adjust protein synthesis rates as a way to swiftly adapt to external perturbations, which can alter cell fate towards differentiation^42^. We and others have previously found that a diverse range of tissue stem cells share a particular degree of sensitivity to ER stress and activation of a UPR mediated, PERK-eIF2α dependent, global translational arrest^6,9,10^. From an evolutionary perspective, detection of stress in the ER caused by protein misfolding could serve to guard the fitness of the stem cell pool by forcing loss of self-renewal capacity of damaged stem cells. Interestingly, this could be a key mechanism to protect stem cells from accumulation of DNA damage. Research on protein folding in organisms such as yeast and *E. coli* has established that most proteins are encoded in an optimal folding state and that point mutations in protein encoding DNA often result in protein folding difficulties and activation of the UPR^43–45^. Thus, the same carcinogens which cause the oncogenic mutations that promote stem cell survival and growth are likely to generate additional mutations that result in misfolding of ER targeted proteins, eliciting ER stress.

Potential mechanisms that underlie the relationship between protein translation and stem cell differentiation remain poorly characterized. Interestingly, many transcription factors that determine ‘stemness’ including several of the pluripotency factors have a remarkably short protein half-life. Half-life of c-MYC for instance is less than 30 min^46^ and the half-life of Nanog and SOX-2 is 1–2 h^47^. This implies that a brief translational pause could result in the depletion of such factors which in turn will reduce self-renewal capacity. Here, by means of an unbiased proteomic approach, we screen for transcription factors that are activated or inactivated during the early ER stress response and identify a large set of transcription factors from various families, including several co-regulators that can indirectly mediate DNA-binding of multiple transcription factors. To the best of our knowledge, the role of CtBP2 in the regulation of intestinal epithelial stemness and ER stress induced differentiation has not been fully addressed. We found that CtBP2 as a transcriptional regulator not only loses its binding to the catTFRE template DNA, but is also rapidly depleted at the protein level by ER stress in a manner that is PERK dependent. *CtBP2* mRNA expression levels were found to be gradually distributed over the crypt-villus axis, whereas protein expression was more restricted to the crypt region, suggesting post-transcriptional regulation expression. Strikingly, CtBP2 overexpression alone was sufficient to tip the balance between stemness and differentiation, leading to enrichment and increased clonogenicity of stem cells and reduced stem cell differentiation in epithelial organoids. Upregulated CtBP2 was identified at the earliest stage of colorectal carcinogenesis when loss of APC and hyperactivated WNT signaling occurs, events that drive proliferation and de-differentiation^48^. The crucial role for CtBP2 in the clonogenic capacity of APC and APC-KRAS mutated cells that we observe, is in line with previous findings where *Ctbp2* heterozygosity in *Apc*^Min/+^ mice restricted the population of tumor initiating cells and the number of developing polyps as a result^49^.

High expression of *CtBP2* and the downstream target *TIAM1* in human CRC correlated with a poor prognosis, and overexpression in intestinal epithelial cells protected against radiation-induced injury and ER stress-induced loss of stemness. Based on these results, it is tempting to speculate that tumor cells with stabilized CtBP2 expression are selected for over time, because of their ability to overcome impaired self-renewal imposed on stem cells by oncogenic stress conditions such as DNA damage or proteotoxicity. The observed CtBP2-mediated upregulation of BMI1 in the radioresistant CtBP2 overexpression organoids, could play a role in resistance of cancer cells with elevated CtBP2 to therapeutic chemo-radiation. Indeed, BMI1 expressing intestinal stem cells are known to be more resistant to high-dose radiation injury since BMI facilitates the regenerative response upon DNA-damage^50^. A similar mechanism has been reported in neural stem cells^51^. Finally, we should mention that the function of CtBP2 in the context of the UPR as mediator of apoptosis was not investigated in this study, and could be a potential mechanism through which CtBP2 extends its oncogenic properties. It is interesting to note that CtBP2 has been linked to JNK signaling, given that its downstream transcription factor c-JUN was significantly altered in our proteomic screen^52^.

In conclusion, we exposed a set of transcription factors with lower DNA-binding activity during the early phase of ER stress. CtBP2 was further examined and found to be sufficient and required for proper stem cell fate, response to cellular damage and oncogenic expansion, features that potentially characterize CtBP2 as an oncoprotein. Although no mutations have been described in the gene, there is a selective pressure to enrich for CtBP2 expressing cells as it binds to truncated APC in FAP patients, promoting oligomerization and thereby inhibiting ß-catenin degradation^53^. Future research will tell whether targeting CtBP2 might be feasible in CRC patients, a strategy that may be particularly interesting for FAP patients.

## Material and methods

### Ethics statement

Written informed consent was obtained from all donors in this study for the use of the material for research purposes. Tissues were obtained with approval of the ethical committee of the Amsterdam UMC, together with approval of the experimental procedures by the HIS Mouse Facility (Amsterdam UMC). All methods were performed in accordance with the relevant guidelines and regulations, as stated in the Amsterdam UMC Research Code, in a certified laboratory (ISO15189 accreditation M304).

### Patient material

Fresh adenoma and adjacent healthy tissue samples were collected and snap-frozen immediately after resection from patients in the endoscopy program for the removal of large (> 1 cm) colorectal adenomas at the University Medical Centers Amsterdam (location AMC). All patients provided written informed consent (METC2015_206). mRNA was isolated from tissue samples by mechanical disruption using the FastPrep-24-5G (MP Biomedicals) in combination with Lysis Tubes S (Qiagen, Cat No./ID: 19091) for 2 times 1 min at 6.5/s with 1 min on ice in between followed by the Allprep DNA/RNA Universal kit (Qiagen, ID 80204).

For generation of human FAP organoids, adenomatous material from the colon was collected at the University Medical Centers Amsterdam (location AMC), after being approved by the Medical Ethical Committee (normal tissue: MEC 09/146 and MEC 05/071).

### Animal experiments

The study was carried out in compliance with the ARRIVE guidelines. All animal experimentation methods were performed in accordance with the Animal Ethical Committee guidelines and regulations of the Leiden University Medical Center (*Workprotocol 68215*), the Netherlands. For ER stress induction, adult wild‐type (WT) C57Bl/6J mice received a single intraperitoneal injection of thapsigargin dissolved in DMSO (1 mg/kg) or DMSO alone 8 h or 24 h before being euthanized.

For CtBP2 immunostaining in homeostatic stem cells, and immunostaining in *APC*-driven polyps, the *Lgr5*-EGFP-IRES-creERT2 knock-in mouse and the *Villin*-Cre-ERT2-*Apc*^15lox/+^ mouse models were used, respectively.

### Cell culture, organoid culture and transduction

LS174T colorectal cancer cells (ATCC CL188) were grown in Dulbecco’s modified Eagle’s medium with 10% fetal calf serum (FCS) and 1% penicillin/streptomycin under standard culture conditions.

Mouse small intestinal epithelial organoids were cultured in ENR medium containing N2, B27 supplements (Invitrogen), 1.25 mM n-acetylcysteine, 50 ng/ml mouse EGF (Invitrogen), Noggin-Fc-conditioned medium (20%, equivalent to 200 ng/ml), Rspo1-Fc-conditioned medium, unless specifically stated otherwise. Patient-derived FAP organoids were cultured in ENR medium, with addition of 2-mercaptoethanol (Sigma-Aldrich), gentamicin (BioWhittaker), 50 mM TGF-βRI inhibitor A38-01 (Tocris Bioscience), 30 mM p38 inhibitor SB202190 (Sigma-Aldrich), and 500 µM (Leu15)-Gastrin (Sigma-Aldrich). Organoid growth was assessed after re-passaging organoids in a 48-well plate and photos were taken using a Leica DM6000 Digital Microscope equipped with LAS AF Software (Leica, Wetzler, Germany). Quantification was done by measuring the perimeter and surface area using Image-J software. For the irradiation-induced regenerative response experiments, we treated the organoid cultures, 3 days after seeding, with 6 Gy of γ-radiation as previously described^54^. We made used of the following organoid models:

*Inducible CtBP2 overexpression* was generated as follows: in short, small intestinal tissue ~ 2 cm was collected from body wide transgenic CAG-rtTA3 mice, which were gently scraped with coverslip, washed with ice-cold PBS, and then incubated with 2 mM EDTA in PBS on a rotating wheel to obtain the epithelial fraction. Residual debris were removed by gentle shaking, the debris-containing supernatant was removed and replaced with cold PBS. This procedure was repeated until supernatant was clear. After passing the supernatant through a cell strainer and centrifuge, the pellet was resuspended in 20 µl Matrigel (BD Biosciences) at a desired crypt density and plated in a 48-wells plate. After expansion of the culture, organoids were prepared for transduction according to a previously described methodology^25^.We used the pRetroX-Tight-Pur vector (Clontech), for expressing a gene with the Tet-On Advanced system, in which we cloned the human coding sequence of CtBP2. After puromycin selection, induction of CtBP2 was achieved using 1 μg/ml doxycycline in ENR medium.

*CtBP2 knockdown in human FAP organoids* was generated as follows: two subclones of polyps from a FAP patient were used, from the organoid biobank at the Amsterdam UMC according to previously described methodology^25^. The first line carried only *APC* loss of heterozygosity, whereas the second line carried an additional spontaneous gain-of-function point mutation in *KRAS* (G > D). Stable knockdown was achieved using lentiviral transduction with the pLKO.1 vector containing 3 different shRNAs against human *CTBP2* (TRCN0000013743, TRCN0000013744 and TRCN0000013746) Selection was ensured using 2 μg/ml puromycin. *CTBP2* after loss of *APC* was studied used *Villin*-Cre-ERT2-*Apc*^15lox/15lox^ organoids that were recombined in vitro using 0.75 µg/ml 4-hydroxy tamoxifen for 24 h.

### CatTFRE assay for transcription factor binding

We used the catTFRE assay to quantitatively measure proteome-wide changes in DNA binding activity of transcription factors in response to ER stress. A detailed methodological description of the catTFRE assay has been described earlier^17^. In brief, LS174T cells were plated in a 6-well plate for treatment, after which cell pellets were harvested and the nuclear fraction was extracted. Subsequently, a pulldown was performed using a biotin-labeled synthetic DNA plasmid containing a concatenated tandem array of consensus transcription factor response elements (TFREs) for most known transcription factor families. After trypsin digestion, the tryptic peptides containing the enriched endogenous transcription factors were subjected to a LTQ-Orbitrap Velos mass spectrometry (Thermo Fisher Scientific) for measurement. Peptides of transcription factors with good signal response were selected for quantification by calculating the area under the curve. Proteins were identified by using the National Center for Biotechnology Information search engine against the human or mouse National Center for Biotechnology Information RefSeq protein databases. The list of candidate genes was further analyzed using DAVID Functional Annotation Tool (david.ncifcrf.gov). We assessed the top 100 genes present in both treatment groups (thapsigargin and Subtilase AB) and looked for significant enrichment of these genes in gene annotation categories of the DAVID database, setting a threshold of minimally 10 genes per category.

### RNA extraction and RT-qPCR analysis

All cells and organoids were harvested at the indicated times, and RNA was extracted using the ISOLATE II RNA Mini Kit (BIO-52073, Bioline). Complementary DNA was synthesized from 550 ng mRNA using Oligo-dT (Thermo Fisher Scientific) and random hexamer primers (Promega, Madison, USA), RiboLock RNase Inhibitor and RevertAid reverse transcriptase (Thermo Fisher Scientific). Quantitative RT-PCR (qPCR) was performed on a CFX96TM Real-Time System (Bio-Rad Laboratories, Hercules, CA, USA) using sensifast SYBR No-ROX Kit (GC-biotech, Bio-98020). The geometric mean expression the reference genes ACTB, HPRT, and GAPDH were used to normalize mRNA levels. All primers used in this study are listed in Table 1.

**Table 1:** Primer sequences for RT-qPCR.

	Forward primer	Reverse primer
**Gene (mus musculus)**		
*Ctbp2*	TCAAGGAGGGCAGGATACGA	ACCAAGGAGCTGAAGTCACG
*Tiam1*	GAAGCACACTTCACGCTCC	CTCCAGGCCATTTTCAGCCA
*Lgr5*	TGTGTCAAAGCATTTCCAGC	CAGCGTCTTCACCTCCTACC
*Olfm4*	AACATCACCCCAGGCTACAG	TGTCCACAGACCCAGTGAAA
*Ascl2*	GAAGGTGCAAACGTCCACTT	TCCATCAAGCTTGCATTCAG
*Bmi1*	TGATTCTGGTTGTTCGATGC	TGGCTCGCATTCATTTTATG
*Axin2*	GAGAGTGAGCGGCAGAGC	CGGCTGACTCGTTCTCCT
*Lyz1*	ATGGAATGGCTGGCTACTATGG	ACCAGTATCGGCTATTGATCTGA
*Muc2*	GAAGCCAGATCCCGAAACCA	GAATCGGTAGACATCGCCGT
*Alpi*	CACAGCTTACCTGGCACTGA	GGTCTCTGACGACAGGGGTA
*Grp78*	TGGCACTATTGCTGGACTGA	TTCAGCTGTCACTCGGAGAA
**Genes (homo sapiens)**		
*CTBP2*	AGTTCAAGGCCCTGAGAGTGAT	CATAGGTTGAGGATGTGGCAGAT
*TIAM1*	GATCCACAGGAACTCCGAAGT	GCTCCCGAAGTCTTCTAGGGT
*LGR5*	CGGAGGAAGCGCTACAGAAT	CTGGGTGGCACGTAGCTGAT
*OLFM4*	CTCCATGATGTCAATTCGGA	CAGAGTGGAACGCTTGGAAT
*CD44*	TGCCGCTTTGCAGGTGTAT	GGCCTCCGTCCGAGAGA
*LRIG1*	CTGGACGCGGAGCCTAAAC	TGTAGGTTCGGCAAGTCCTCA
*CDKN1A*	AGTCAGTTCCTTGTGGAGCC	CATGGGTTCTGACGGACAT
*MUC2*	GAAGCCAGATCCCGAAACCA	GAATCGGTAGACATCGCCGT
*DNAJB11*	GGATCTGGGTGCTGCTTATG	TGTCTCCATGGGAGCTCTG
*DNAJC3*	ACAAGGAAAACTTGATGAAGCAG	TGAGACTGTGCTTCCTTTTCTTC
*DNAJB9*	CAGCTCTTGTGGAGGAGCAG	AATGCAGATTGCAAAGATGAAA
*EDEM1*	GCTCAACCCCATCCACTG	CCAATGCATCAACAAGAGTCA

### Immunoblots

Cells were lysed in cell lysis buffer (Cell Signaling Technology), and heated to 95 °C for 5 min in sample buffer containing 0.25 M Tris-HCl (pH 6.8), 8% SDS, 30% glycerol, 0.02% bromophenol blue and 1% β-mercaptoethanol. Separation was done on 6–12% SDS-PAGE, and proteins were transferred to a PVDF membrane. Specific detection was done by incubating the blot overnight in TBS with 0.1% Tween-20 and 1% BSA. Antibody binding was visualized using the Lumi-Light western blotting substrate (Roche). Images were captured with a Leica DM6000 microscope using LAS AF software (Leica, Wetzlar, Germany). All western blot images were generated and exported using ImageQuant LAS 4000 software and cropped and labeled using Adobe Illustrator software version 25.2. Antibodies used: anti-CTBP2 (Cell Signaling, #13256), anti-ZMYM2 (Abcam, ab106624), anti-FOXO3 (Cell Signaling, #2497s), anti-ZFPM1 (Abcam, ab86281), anti-CDX1 (Abcam, ab126748), anti-MNX1 (Abcam, ab95926), anti-IRF8 (Cell Signaling, #5628), anti-c-MYC (Santa Cruz, sc-764), anti-CD44v6 (Biorad, #MCA1967), anti-TCF4 (Cell signaling, #2569S) anti-GRP78 (Cell Signaling #3183), anti-PERK (Cell Signaling #3192), anti-beta Actin (Sigma-Aldrich, A1978), Vinculin (Cell Signaling, #18799S).

*L-Azidohomoalanine (AHA) incorporation and pulldown assays* were performed to quantify nascent protein levels of CtBP2. In brief, endogenous methionine was depleted in LS174T cells by preincubation in methionine and cysteine free DMEM supplemented with glucose and glutamine. Subsequently, cells were labeled with 1 mM of AHA (Iris Biotech) and 400 nM of thapsigargin for 4 h, and controls were added for labeling (50/50 ratio of methionine and AHA) and global translation (100 mM cycloheximide). Then, after 2 washes with HBSS (w/o calcium) protein lysate was harvested in HEPES buffer (pH 7.4) containing 1% NP-40 with protease inhibitors. After measuring protein content using BCA, a click reaction was achieved by adding a mixture of the following components per 500 µg protein: 250 µM biotin-alkyne (Sigma-Aldrich), 1 mM copper sulphate (Sigma-Aldrich), 5 mM THPTA (Sigma-Aldrich) and 15 mM sodium ascorbate (Sigma-Aldrich). After incubate at room temperature for 1 h on the end-over-end shaker, 2 µl of 0.5 M EDTA was added to each sample, and lysates were loaded on a Zeba column to remove unbound Dde-alkyne biotin. 40 µl of washed NeutrAvidin beaded agarose resin (Thermo Fisher Scientific) in a 50/50 slurry with PBS was added and samples were incubated overnight on an end-over-end rotor at 4 degrees. After several washes in PBS + 1%NP40, Laemmli sample buffer supplemented with 100 mM DTT was added, following 15 min incubation at 65 degrees. This process was repeated in a 10 min incubation at 90°, after which samples were loaded on SDS-PAGE, proceeding with standard Western blot procedure (see above).

### Immunohistochemistry

Tissue was fixed in 10% ice-cold formalin and embedded in paraffin^6^. Sections of 4 µm thickness were deparaffinized in xylene and rehydrated. Endogenous peroxidase was blocked using 0.3% H_2_O_2_ in methanol for 30 min. The following methods of antigen retrieval were used: Sodium citrate (slides were cooked at 100 °C for 20 min in 0.01 M sodium citrate, pH 6); Tris/EDTA (slides were cooked at 100 °C for 20 min in a Tris/EDTA buffer, 10 mM Tris, 1 mM EDTA, pH 9.0); proteinase K (slides were incubated with Proteinase K (Dako, S302030) for 5 min at room temperature). After antigen retrieval slides were blocked in PBS using 0.1% Triton X-100 and 1% bovine serum albumin (PBT) for 30 min, followed by incubation overnight at 4 °C with a primary antibody (anti-CTBP2 Cell Signaling, #13256) in PBT. Antibody binding was visualized using Powervision HRP (Immunologic) labeled secondary antibodies and diaminobenzidine (Sigma-Aldrich) for substrate development. All sections were counterstained with Mayer’s haematoxylin.

*For Immunofluorescence,* slides were incubated for 1 h using fluorescently labeled secondary antibodies (all Alexa Fluor secondary antibodies from Invitrogen), diluted 1:500 in PBS with 0.1% Triton X-100 and 1% bovine serum albumin at room temperature. Slides were washed and mounted with Slowfade Gold Antifade reagent with DAPI (Invitrogen, s36938). Images were obtained on a Leica DM6000 Digital Microscope equipped with LAS AF Software (Leica, Wetzler, Germany). For analysis ImageJ software was used.

*RNAscope in situ hybridization* (ISH) was performed according to the manufacturers’ protocol (Advanced Cell Diagnositcs, ACD). The single-plex RNAscope probe against mouse Ctbp2 (#489981 ACD Europe SRL) was used.

*For migration Scratch assays* LS174T cells were grown in a 6-well plate until full confluence was reached. Two scratches per well were made using a p10 pipet tip. Cells migration was filmed overnight with a Leica DMi8 inverted microscope, fitted with a humidified culture chamber maintained at 37 °C and the covered areas were quantified with Image-J software (National Institutes of Health, Bethesda, MD).

### EdU incorporation

EdU staining was performed to analyze cell proliferation by flow cytometry using the Click-iT EdU Alexa Fluor 647 kit (Invitrogen). Organoids were incubated with 10 μM EdU for 3 h followed by TrypLE™ mediated cell-dissociation. Cell were fixed for 20 min with 3.7% formaldehyde, washed and incubated with Click-iT reaction cocktail according to manufacturer’s protocol. Fluorescence was quantified on a flow cytometer (LSR Fortessa) and the percentage of EdU-positive cells was calculated with FlowJo 8.0 software.

### Crystal violet

For crystal violet analyses, cells were fixed with 4% paraformaldehyde in PBS for 15 min and subsequently immersed in 5 mg/ml crystal violet (Sigma C3886) in 2% ethanol in H2O.

### Statistics

Statistical analysis was performed using Prism 8.0 (GraphPad Software, La Jolla, CA). All data are presented as mean ± SEM of three independent experiments of technical triplicates unless specifically stated otherwise. Results were analyzed using Student’s t test or one-way ANOVA followed by Bonferroni post-test as indicated throughout the figure legends. In one instance, the Wilcoxon signed-rank test was used to compare paired samples. Differences were considered statistically significant at a *P* value < 0.05.

## Supplementary Information

Supplementary Information 1.

Supplementary Information 2.
