# Supplementary material for: Endoplasmic reticulum stress regulates the intestinal stem cell state through CtBP2
Source: Sci Rep. 2021 May 10;11:9892. doi: 10.1038/s41598-021-89326-w (PMC8111031; doi:10.1038/s41598-021-89326-w)

**Endoplasmic reticulum stress regulates the intestinal stem cell state through CtBP2**

Bartolomeus J Meijer^1^*, Wouter L Smit^1^*, Pim J. Koelink^1^, Barbara F Westendorp^1^, Ruben J de Boer^1^, Jonathan HM van der Meer^1^, Jacqueline Ludovicus Maria Vermeulen^1^, James C Paton^2^, Adrienne W Paton^2^, Jun Qin^3^, Evelien Dekker^4^, Vanesa Muncan^1^, Gijs R van den Brink^1,2,5^, Jarom Heijmans^1,6^**

** Contributed equally*

**Supplementary immunoblotting data**


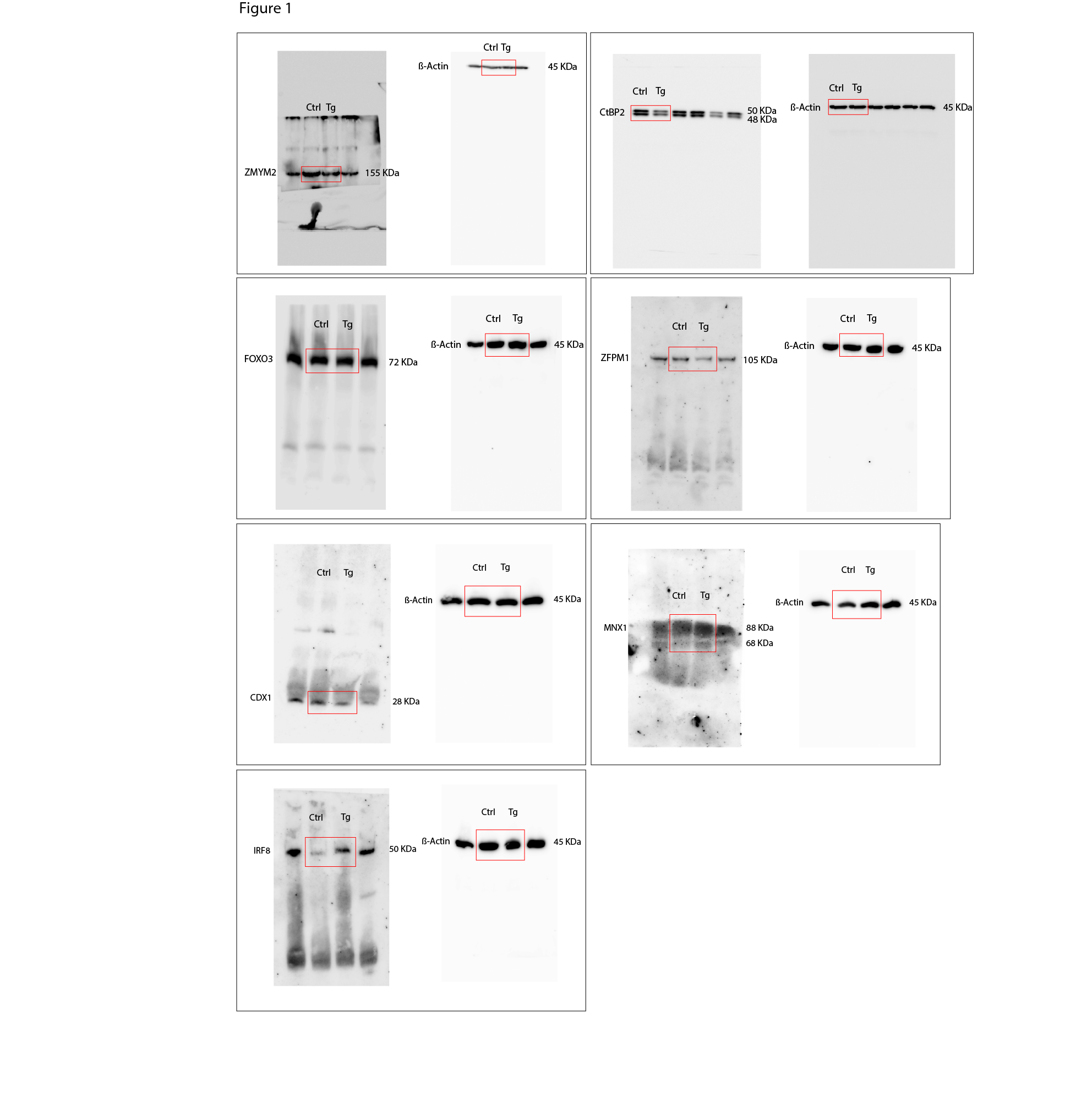

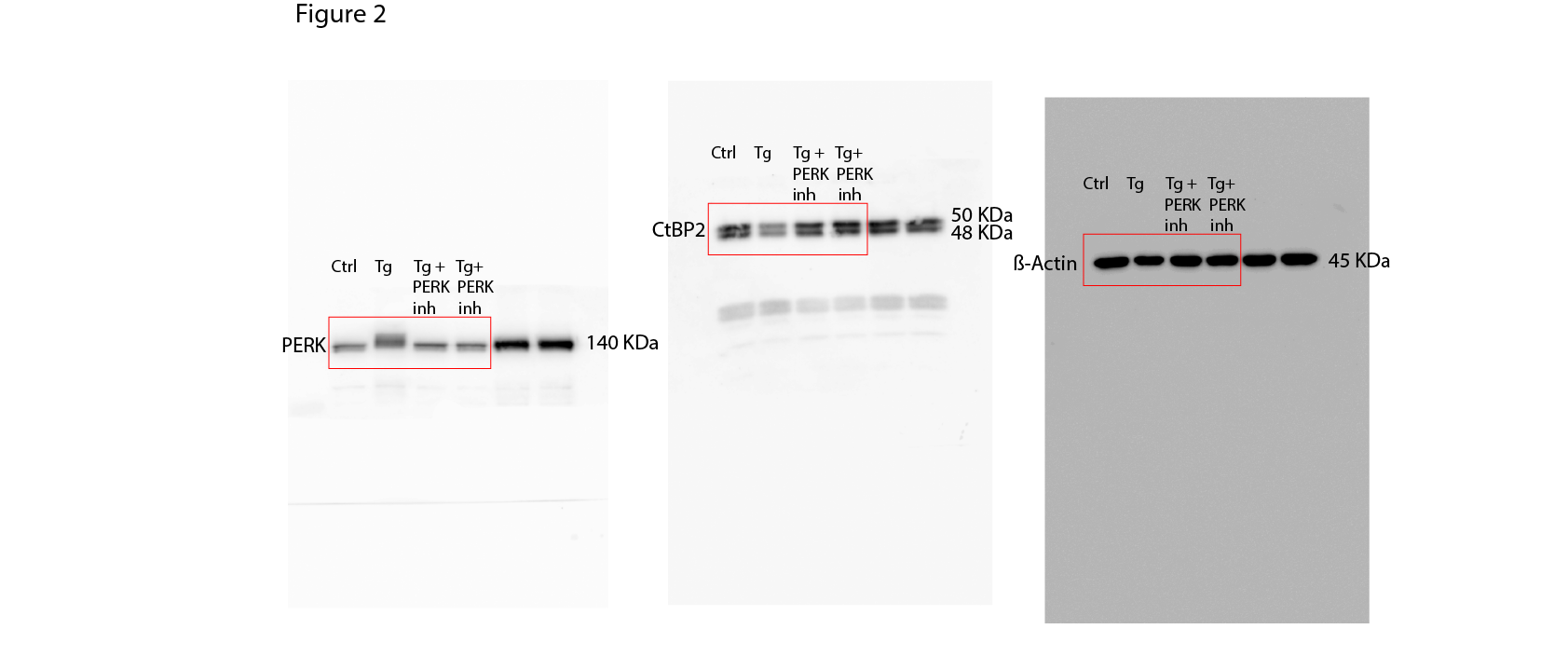


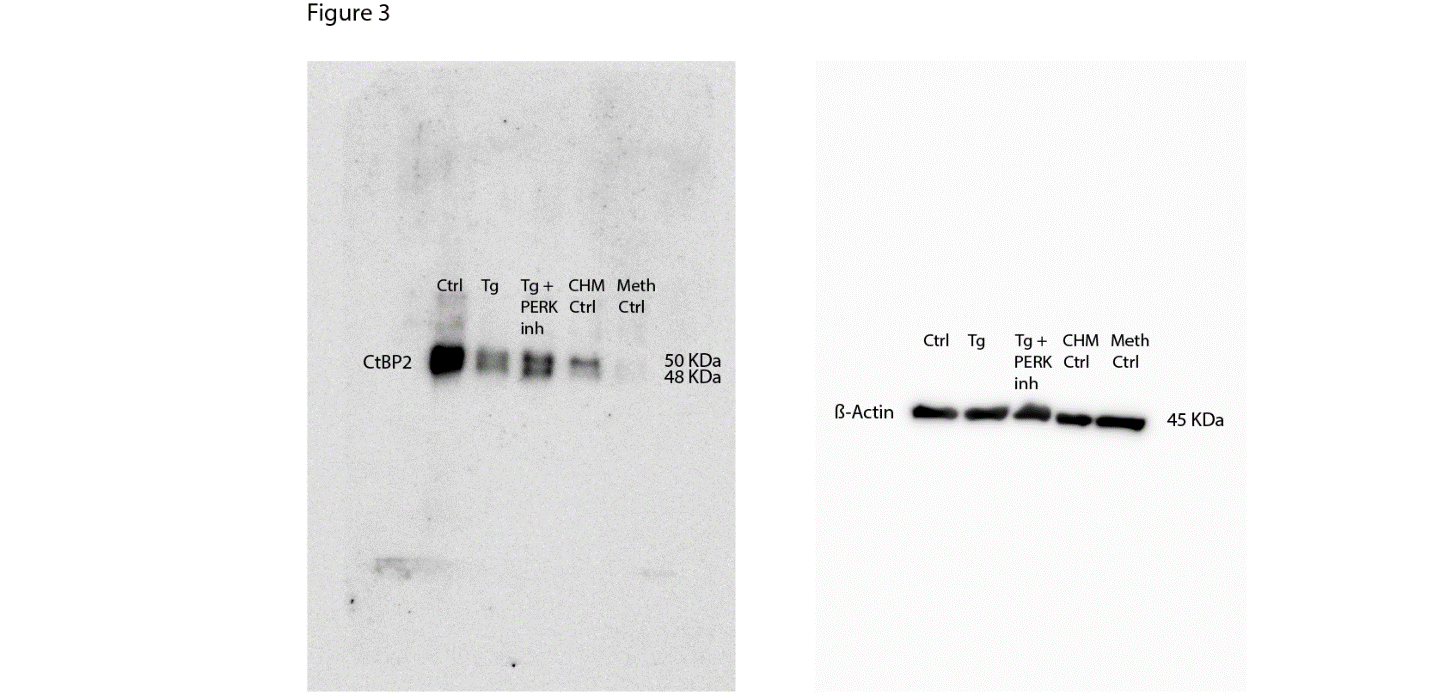


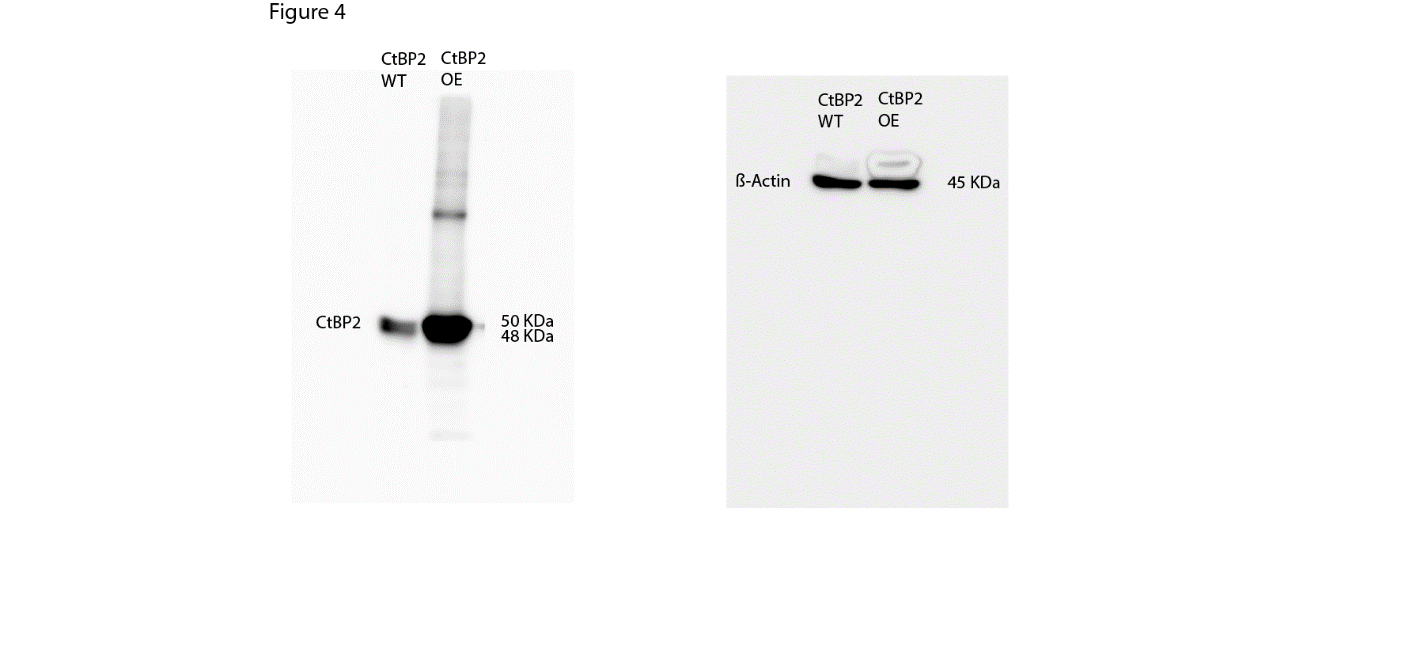


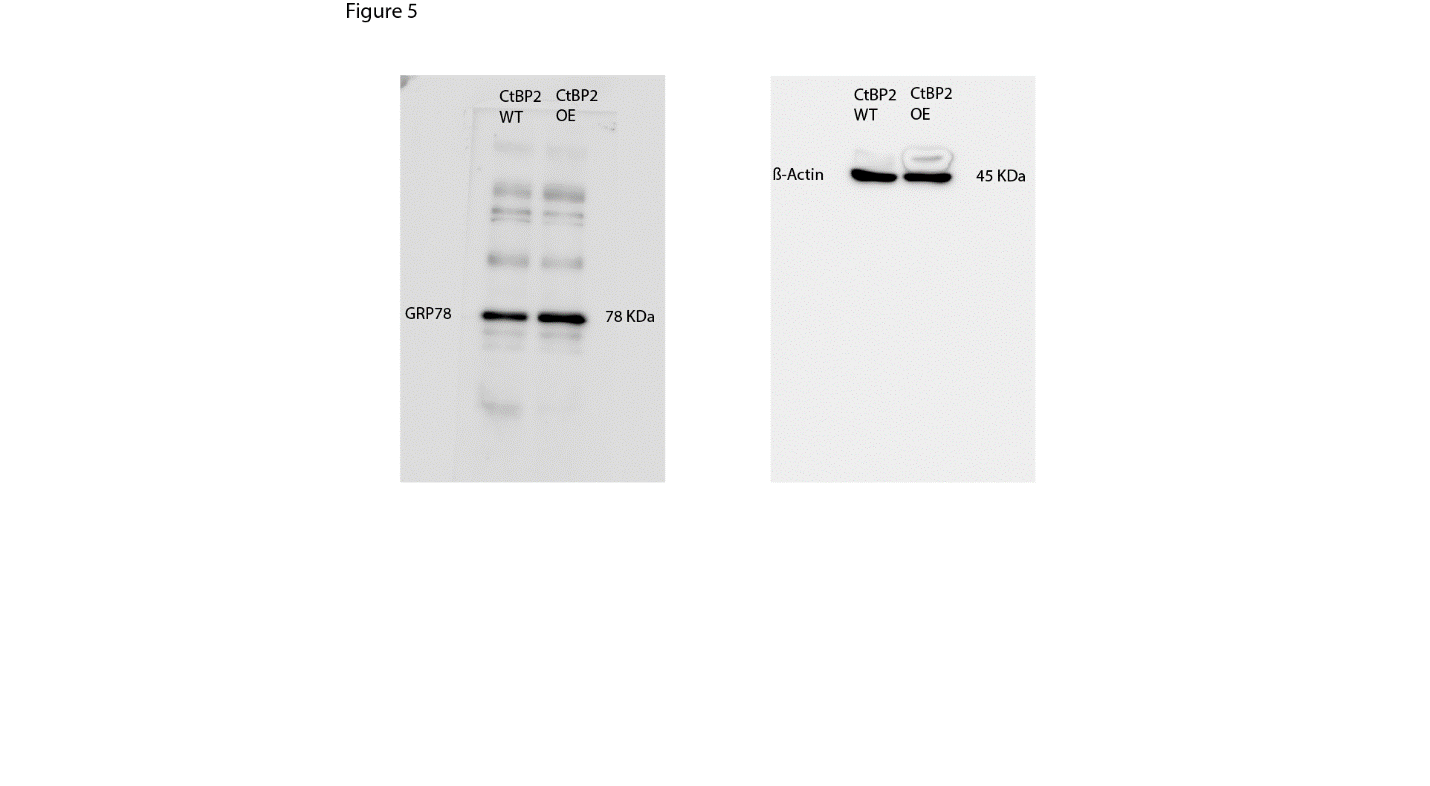


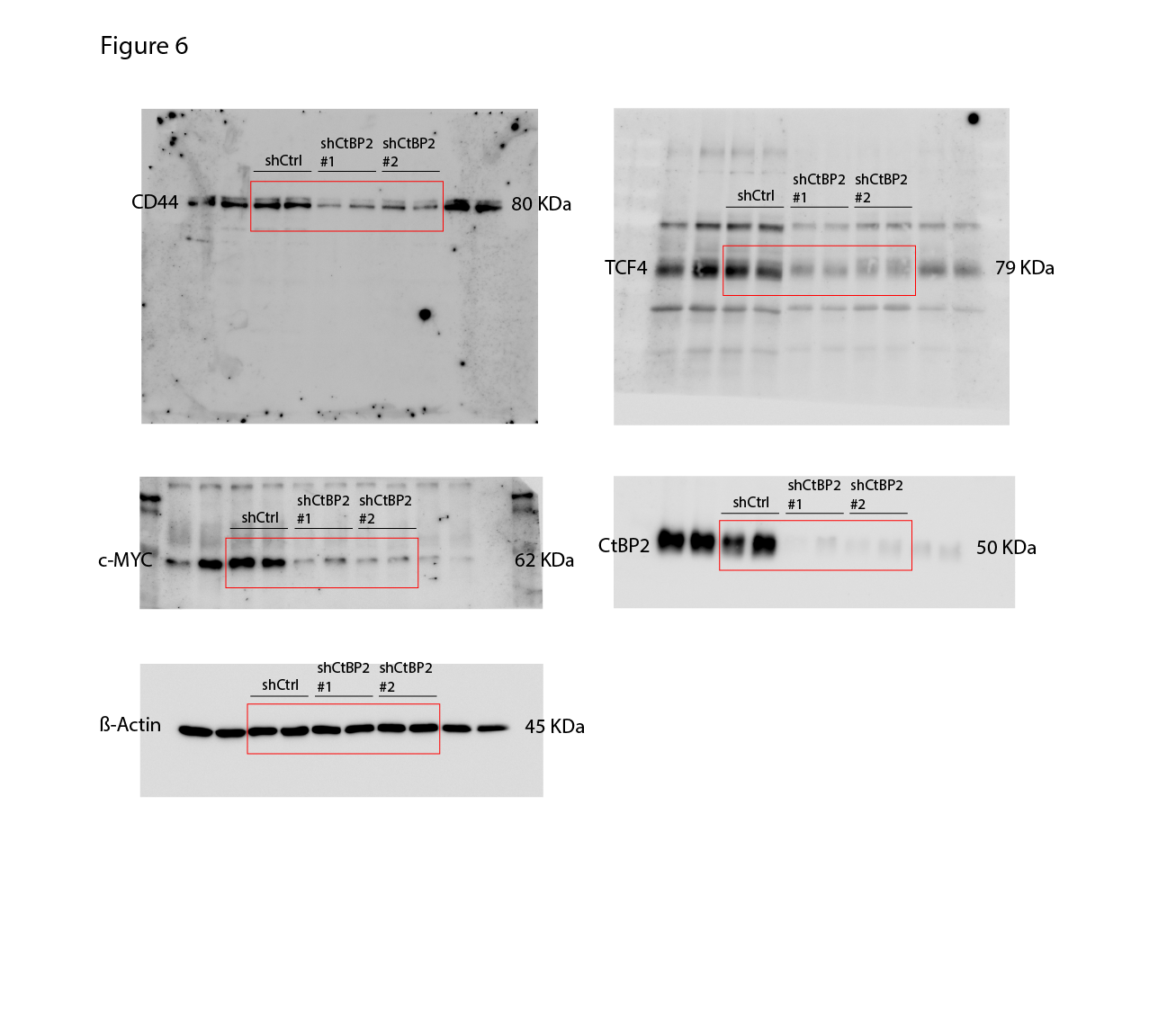

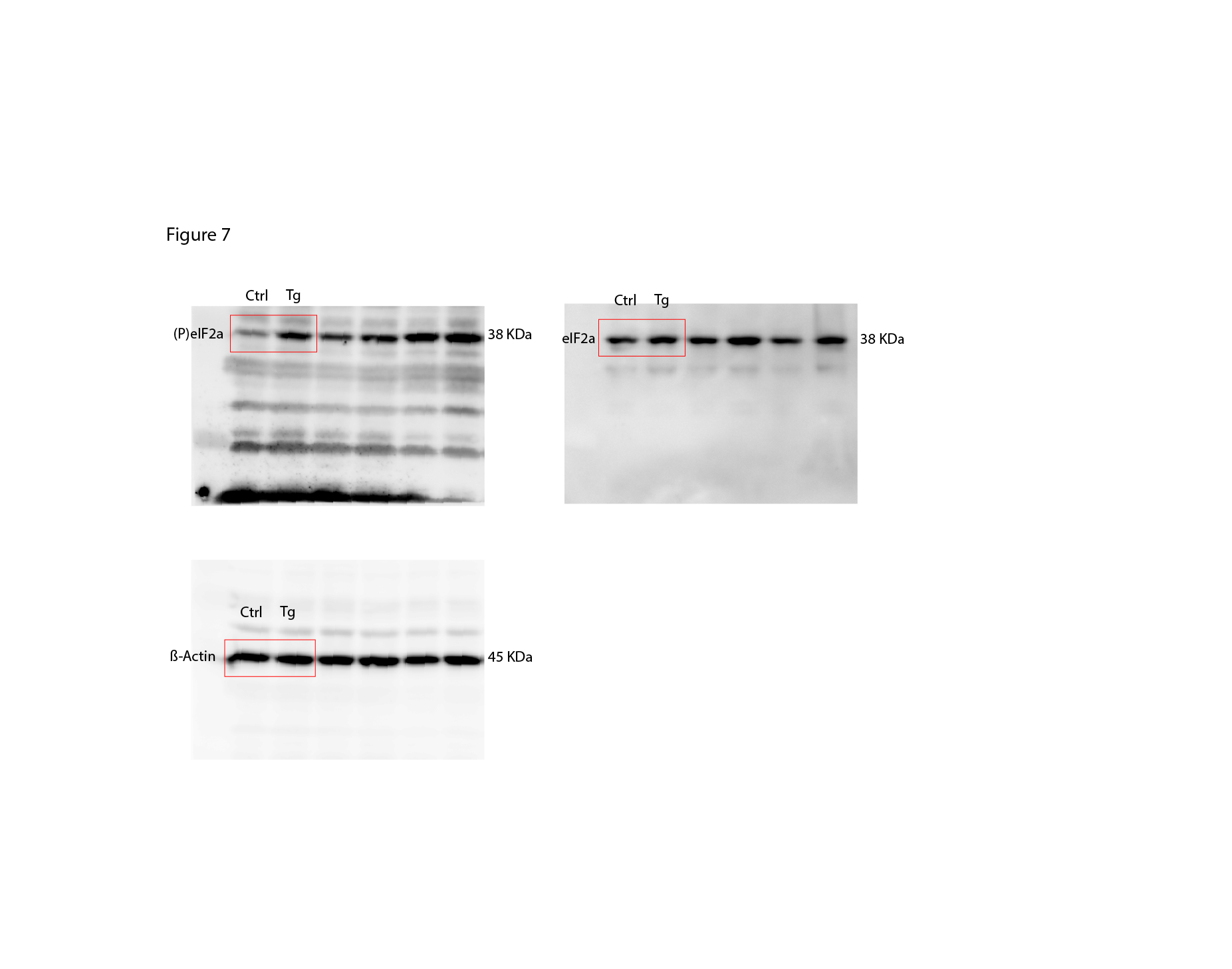


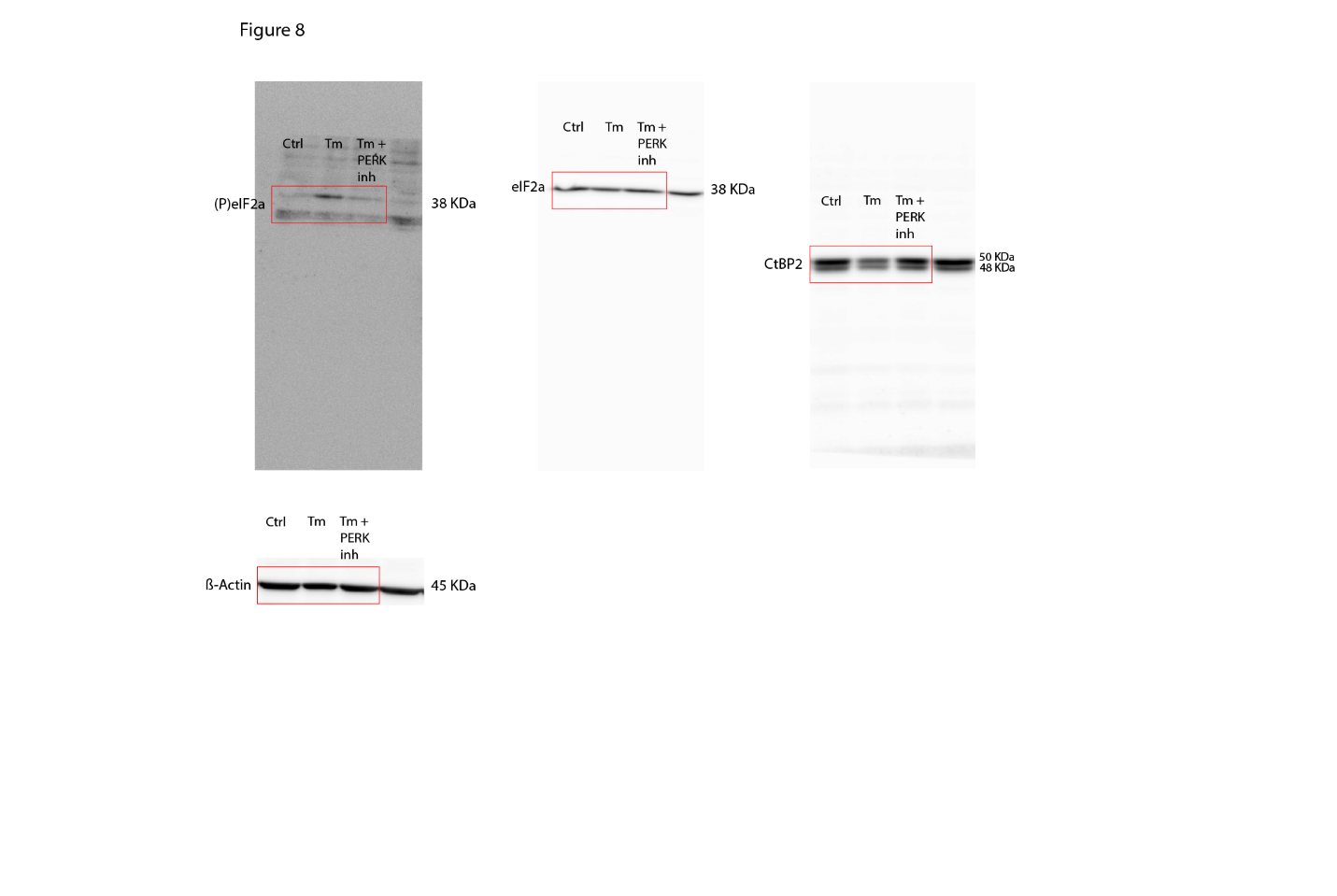


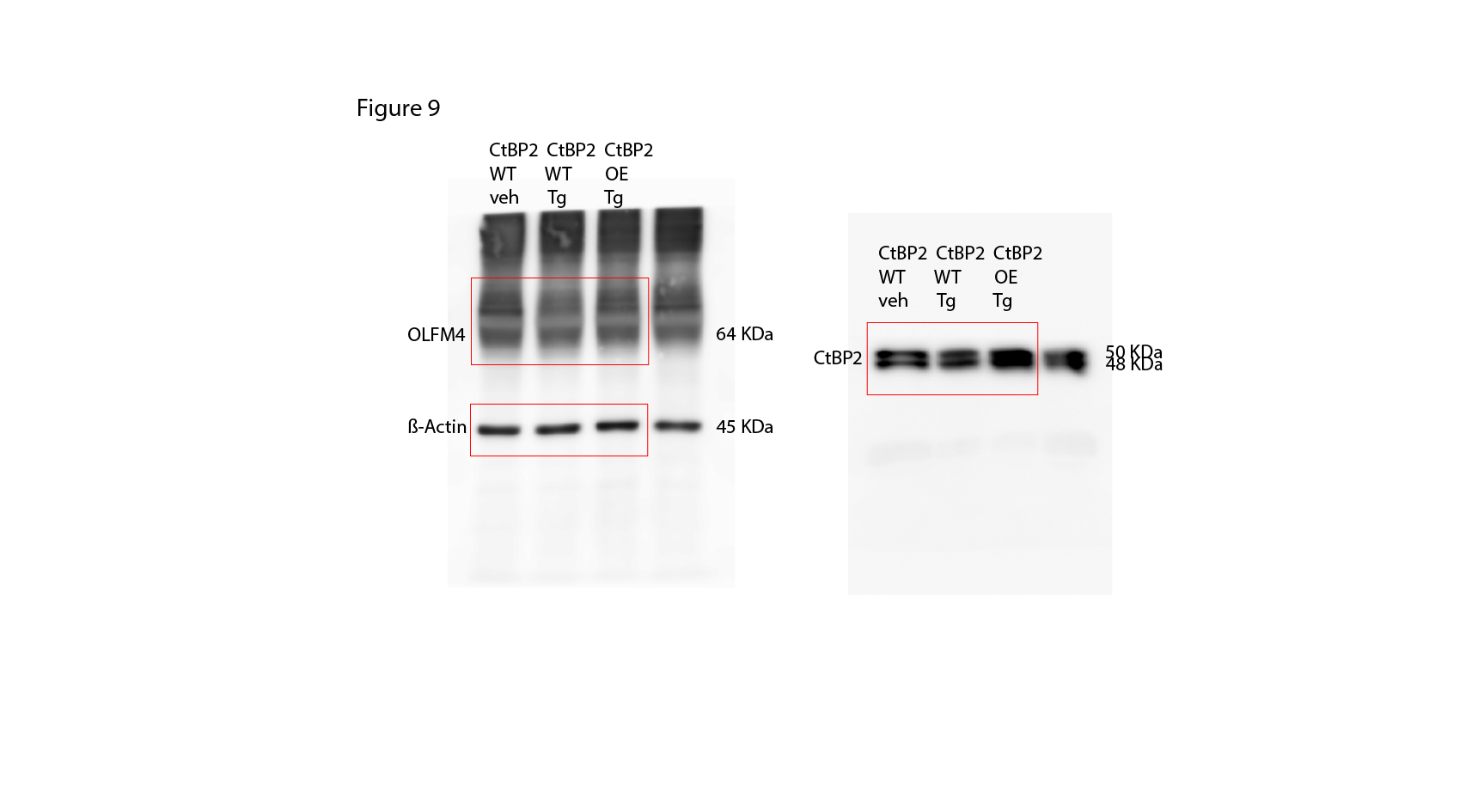

Supplement: Supplementary file 1 — Supplementary Information 1. [file 41598_2021_89326_MOESM1_ESM.docx]
