# Supplementary material for: Endoplasmic reticulum stress regulates the intestinal stem cell state through CtBP2
Source: Sci Rep. 2021 May 10;11:9892. doi: 10.1038/s41598-021-89326-w (PMC8111031; doi:10.1038/s41598-021-89326-w)

**Endoplasmic reticulum stress regulates the intestinal stem cell state through CtBP2**

Bartolomeus J Meijer^1^*, Wouter L Smit^1^*, Pim J. Koelink^1^, Barbara F Westendorp^1^, Ruben J de Boer^1^, Jonathan HM van der Meer^1^, Jacqueline Ludovicus Maria Vermeulen^1^, James C Paton^2^, Adrienne W Paton^2^, Jun Qin^3^, Evelien Dekker^4^, Vanesa Muncan^1^, Gijs R van den Brink^1,2,5^, Jarom Heijmans^1,6^**

** Contributed equally*

**Supplementary figures**

**Figure S1. Experimental setup**

(A) Westerblot analysis showing phosphorylation of eIF2a after 2 hours of thapsigargin treatment in LS174T cells. Full-length blots/gels are presented in Supplementary Immunoblotting Data Figure 7. (B) RT-qPCR of *GRP78, CHOP* and *XBP1(s)* in LS174T cells 2 hours after DMSO or 400nM thapsigargin treatment (n=3). (C) RT-qPCR expression of the downstream target genes of XBP1 and ATF6 signaling *ERDJ4*, *EDEM1*, *P58IPK* and *HEDJ* in LS174T cells following 8 hour treatment with 400nM thapsigargin (n=3). (D) RT-qPCR of *GRP78* and *CHOP* in LS174T cells 2 hours after DMSO or 100 ng/mL Subtilase AB (SubAB) treatment (n=3). (E) RT-qPCR expression of downstream XBP1 and ATF6 target genes *ERDJ4*, *EDEM1*, *P58IPK* and *HEDJ* in LS174T cells following 8 hour treatment of 100 ng/mL SubAB (n=3). (F) Schematic illustration of CatTFRE analysis, adapted from Ding et al. PNAS 2013. Graph bars show mean and s.e.m. * = *P* < 0.05, ** = *P* <0.01, *** = *P* <0.001, **** = *P* <0.0001 (One-way ANOVA). Ctrl = control; Tg = thapsigargin.

**Figure S2. FACS sort strategy for CtBP2 localization**

(A) Small intestinal epithelium from wildtype mice was analyzed by flow cytometry, and cells were gated on EpCAM+CD45- and CD24. Representative histograms of this gating strategy are shown. (B) RT-qPCR analysis of sorted CD45^low^CD24^high^ Paneth cells (contaminated with LGR5 positive stem cells); SC/PC, CD45^low^CD24^med^ Stem cells; SC, and CD45^low^CD24^low^ differentiated cells. DC (n=4).

**Figure S3. *mRNA* expression of *Ctbp2* and *Tiam1* in ER stress induced mouse intestine**

RT-qPCR of *Ctbp2* and (B) RT-qPCR of (A) *Ctbp2* and (B) *Tiam1* in small intestinal epithelium of mice treated for 8 and 24 hours with DMSO or thapsigargin [1mg/kg] dissolved in DMSO (mean expression levels of 5-6 mice per group). (C) RT-qPCR of *TIAM1* expression in LS174T cells treated for 8 hours with SubAB (n=3). (D) Representative western blot analysis of PERK, eIF2a total and phosphorylation, and CtBP2 in LS174T cells treated with tunicamycin [0.5 µg/mL] for 20 hours (n=2). Full-length blots/gels are presented in Supplementary Immunoblotting Data Figure 8. (E) RT-qPCR of total *Xbp1*, spliced *Xbp1* and their ratio in CAG-rtTA-*CtBP2* organoids after 24h overexpression of CtBP2. (F) Representative westernblot analysis of CtBP2 and OLFM4 expression in CAG-rtTA-*CtBP2* organoids treated 6 hours with thapsigargin, with and without 24 hour pre-treatment with doxycycline to induce CtBP2 overexpression (n=2). Full-length blots/gels are presented in Supplementary Immunoblotting Data Figure 9. All western blot images were generated and exported using ImageQuant LAS 4000 software and cropped and labeled using Adobe Illustrator software version 25.2. Graph bars show mean and s.e.m. * = *P* < 0.05, **** = *P* <0.0001 (Student’s *t*-test). Tm = tunicamycin; Tg. = thapsigargin; PERKinh = PERK inhibitor; OE = overexpression.

**Figure S4. Inhibition of proliferation and *TIAM1* expression upon knockdown of *CtBP2* in LS174T cells**

(A) RT-qPCR of *CtBP2* expression showing knockdown in LS174T cells 5 days after transduction with short hairpins against *CtBP2* (*shCtBP2*). (B) EdU incorporation assay after transduction with *shCtBP2* (n=3). (C) RT-qPCR of stem cell markers after transduction with *shCtBP2.* (D) RT-qPCR of differentiation markers upon transduction with *shCtBP2*. (E) RT-qPCR of *TIAM1* upon transduction with *shCtBP2.* (F) Kaplan Meier plot of overall survival separated by *TIAM1* expression in mixed colon adenocarcinoma, from TCGA cohort. (G) *TIAM1* mRNA expression in microarray data from CMS Dataset Guinney et al. Graph bars show mean and s.e.m. * = *P* < 0.05, ** = *P* <0.01 (One-way ANOVA). CMS = consensus molecular subtype.


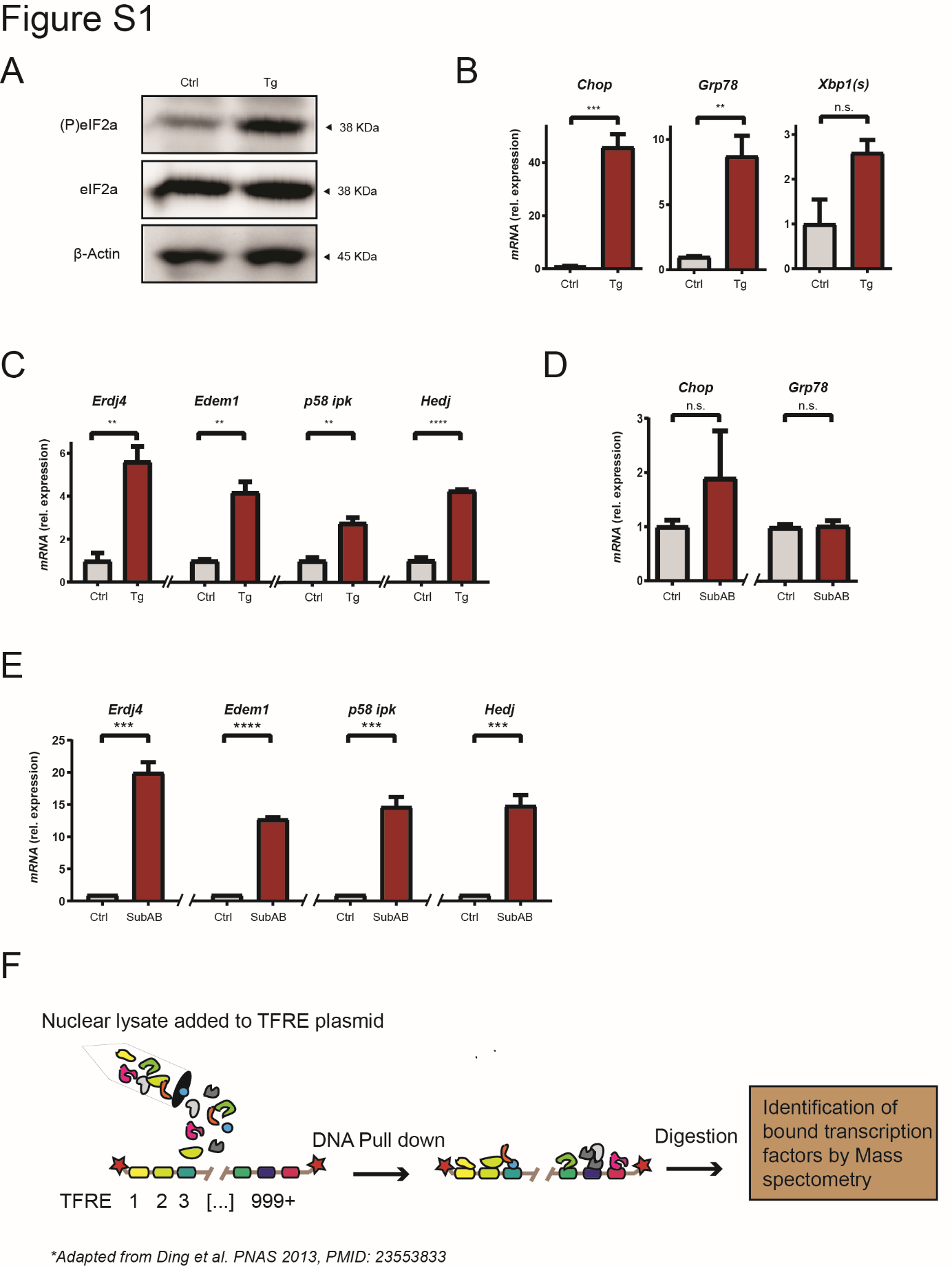


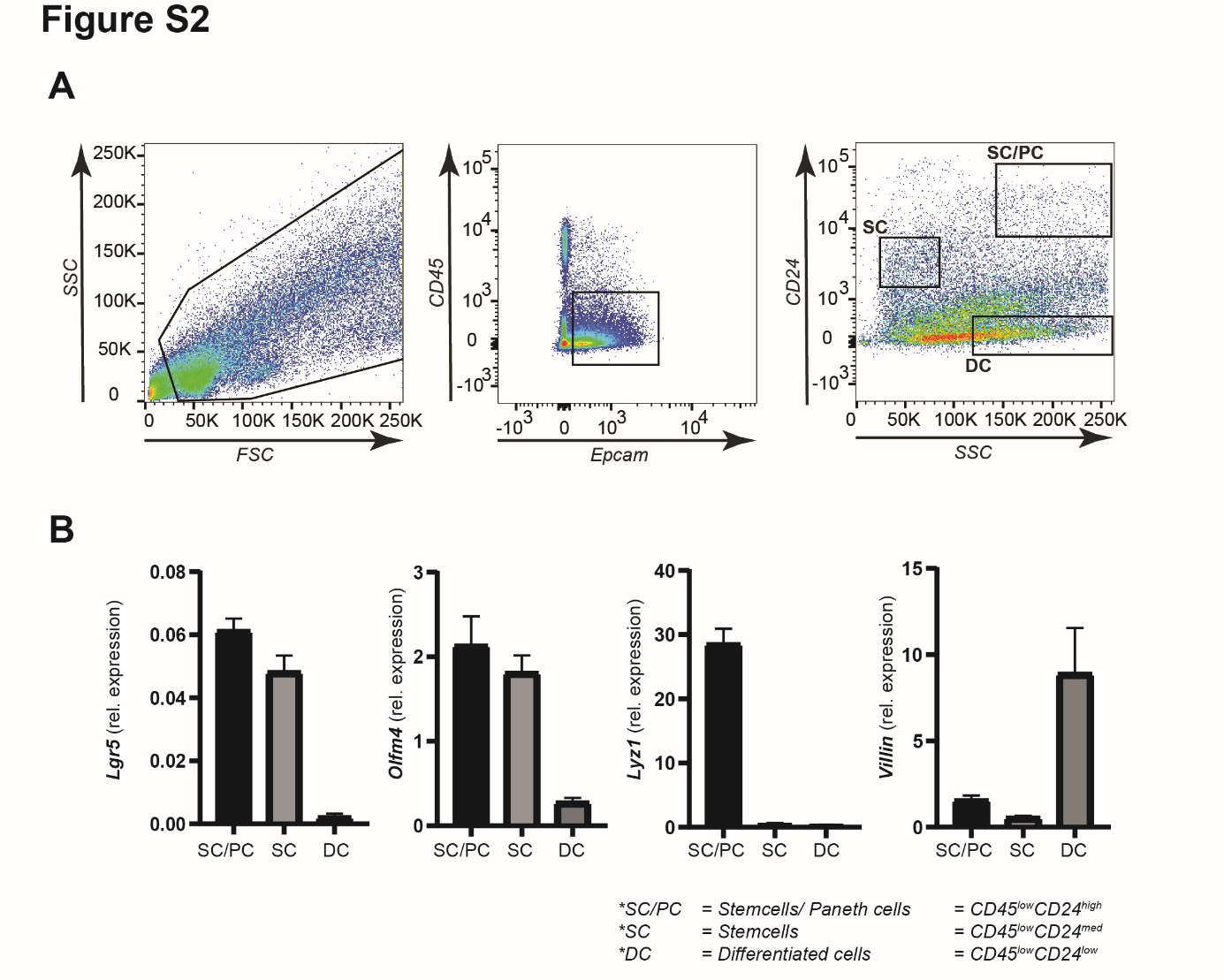


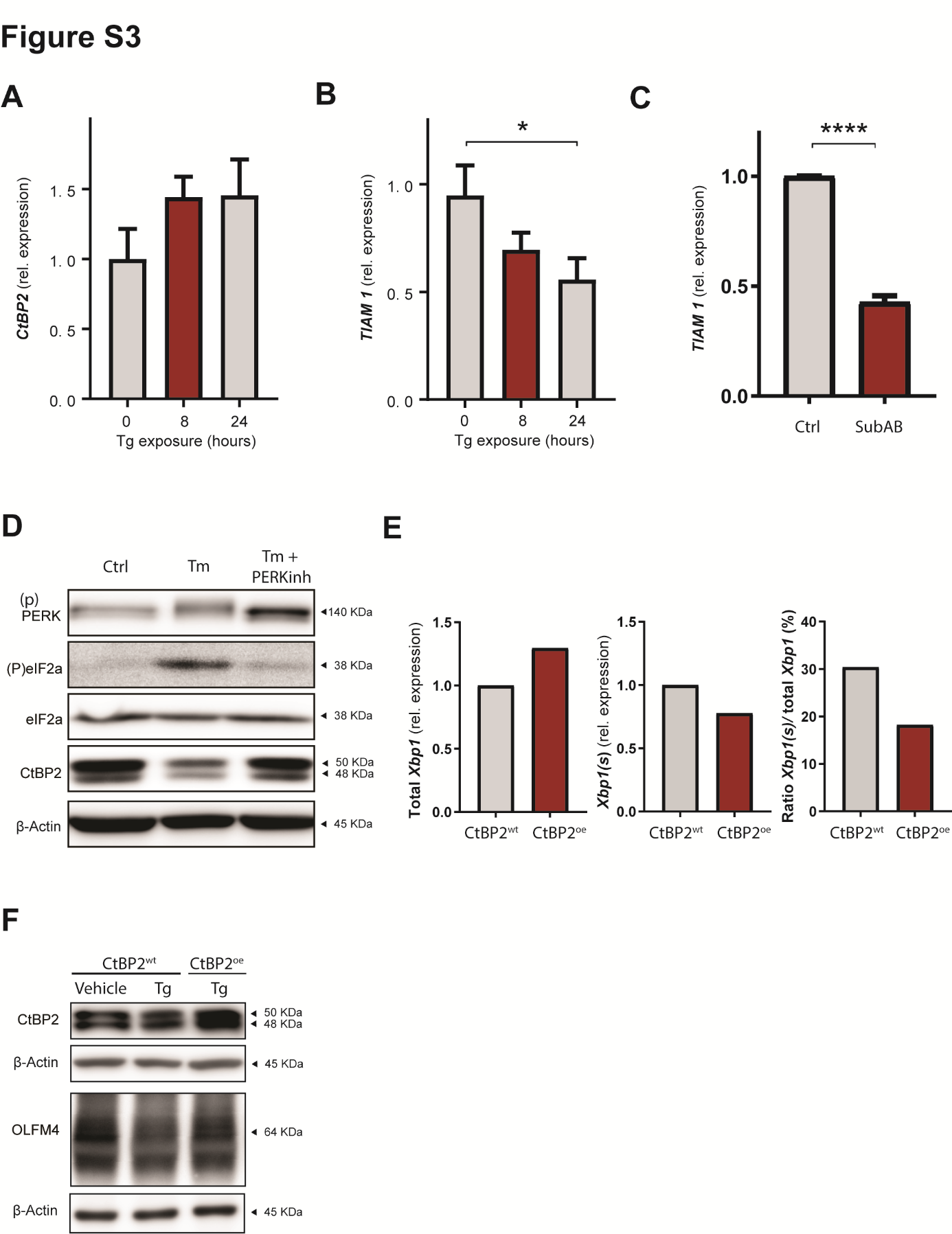


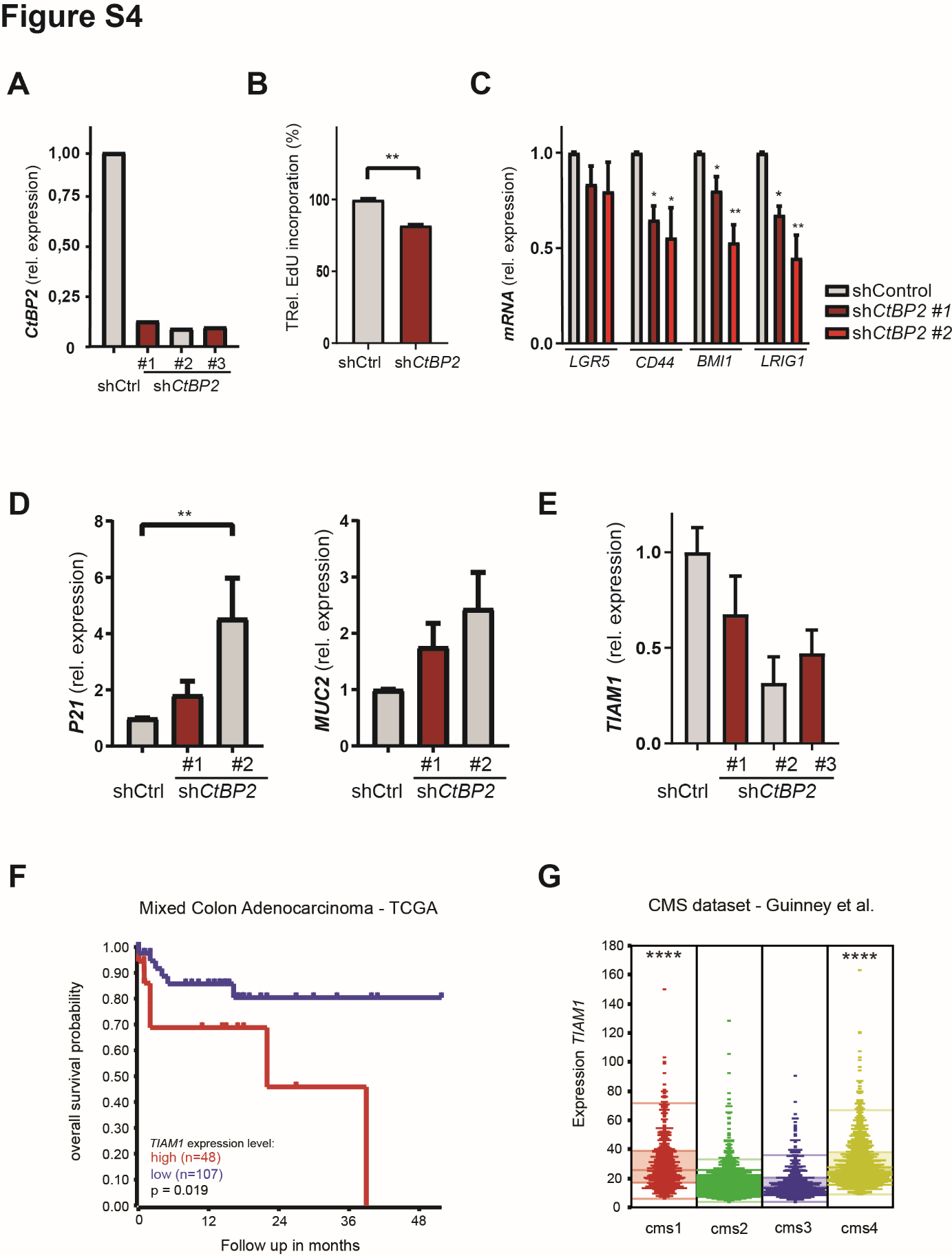

Supplement: Supplementary file 2 — Supplementary Information 2. [file 41598_2021_89326_MOESM2_ESM.docx]
